# CDK6 is essential for mesenchymal stem cell proliferation and adipocyte differentiation

**DOI:** 10.3389/fmolb.2023.1146047

**Published:** 2023-08-16

**Authors:** Alexander J. Hu, Wei Li, Apana Pathak, Guo-Fu Hu, Xiaoli Hou, Stephen R. Farmer, Miaofen G. Hu

**Affiliations:** ^1^ Division of Hematology and Oncology, Tufts Medical Center, Department of Medicine, Boston, MA, United States; ^2^ Department of Surgery, Harvard Medical School, Brigham and Women’s Hospital, Boston, MA, United States; ^3^ National Clinical Research Center of Cancer, Key Laboratory of Cancer Prevention and Therapy, Tianjin Medical University Cancer Institute and Hospital, Tianjin, China; ^4^ Assay Research and Development Department, GRAIL LLC, Menlo Park, CA, United States; ^5^ Center for Analysis and Testing, Zhejiang Chinese Medical University, Hangzhou, China; ^6^ Department of Biochemistry, Boston University School of Medicine, Boston, MA, United States

**Keywords:** Cdk6, stem cells, progenitors, obesity, Runx1

## Abstract

**Background:** Overweight or obesity poses a significant risk of many obesity-related metabolic diseases. Among all the potential new therapies, stem cell-based treatments hold great promise for treating many obesity-related metabolic diseases. However, the mechanisms regulating adipocyte stem cells/progenitors (precursors) are unknown. The aim of this study is to investigate if CDK6 is required for mesenchymal stem cell proliferation and adipocyte differentiation.

**Methods:** Cyclin-dependent kinase 6 (*Cdk6*) mouse models together with stem cells derived from stromal vascular fraction (SVF) or mouse embryonic fibroblasts (MEFs) of *Cdk6* mutant mice were used to determine if CDK6 is required for mesenchymal stem cell proliferation and adipocyte differentiation.

**Results:** We found that mice with a kinase inactive CDK6 mutants (*K43M*) had fewer precursor residents in the SVF of adult white adipose tissue (WAT). Stem cells from the SVF or MEFs of *K43M* mice had defects in proliferation and differentiation into the functional fat cells. In contrast, mice with a constitutively active kinase CDK6 mutant (*R31C*) had the opposite traits. Ablation of RUNX1 in both mature and precursor *K43M* cells, reversed the phenotypes.

**Conclusion:** These results represent a novel role of CDK6 in regulating precursor numbers, proliferation, and differentiation, suggesting a potential pharmacological intervention for using CDK6 inhibitors in the treatment of obesity-related metabolic diseases.

## 1 Introduction

Obesity is an epidemic in the world. According to World Obesity Atlas 2022, if the current trend continues, one billion people globally, including one in five women and one in seven men, will be living with obesity by 2030. Despite the potentially huge market, a truly effective and safe therapy for obesity and associated maladies is lacking. In recent years, cell-based therapies have emerged as alternative approaches (Brooks et al., 2018). Mesenchymal stem cells **(**MSCs) have been shown to have therapeutic efficacy to ameliorate systemic inflammation, hyperglycemia, and insulin resistance induced by T2D (Bhansali et al., 2017; Sun et al., 2017; Bi et al., 2018), providing a new regime for the treatment of T2D. However, a comprehensive understanding of the therapeutic mechanisms of MSCs in T2D remains elusive. It is imperative to understand the molecular pathways linking MSCs to these diseases, and to identify proteins or pathways that may serve as targets for pharmacological interventions.

Obesity is the result of both hypertrophy, an increase in adipocyte size, and hyperplasia, an increase in cell numbers (Hirsch and Batchelor, 1976; Lane and Tang, 2005). Adipocyte hyperplasia arises through adipogenesis, a complex process involving proliferation and differentiation of precursors into mature adipocytes (Rosen et al., 2000). Since mature adipocytes are terminally differentiated postmitotic cells, a constant source of precursor cells must be present in the adult adipose tissues to maintain an appropriate mass. Therefore, regulation of proliferation and differentiation of adipocyte precursors is crucial for the maintenance of fat mass homeostasis.

Many studies have attempted to characterize adipocyte precursors using cell surface markers via flow cytometry analysis. Stem cell antigen-1 (Sca-1^+^) is used extensively as a candidate marker for stem cells from hematopoietic, prostate, dermis, and cardiovascular system (Holmes and Stanford, 2007), as well as for adipocyte progenitors in fat depots (Crossno et al., 2006). Recently, Sca-1^+^CD36^+^ cells have been identified as adipocyte precursors with marked triglyceride (TG) storage capacity (Holmes and Stanford, 2007), while Lin^−^Sca-1^+^CD24^+^CD29^+^CD34^+^ cells are capable of proliferating and differentiating into adipose depot *in vivo* after being injected into the residual fat pads of lipo-dystrophic mice (Rodeheffer et al., 2008). However, the mechanisms controlling adipocyte numbers are unclear at present.

Cyclin-dependent kinase 6 (CDK6) is an important cell cycle regulator and has long been suspected to play an important role in development and tumorigenesis. We have produced both knockout (*Cdk6* ^*−/−*^ or *KO*) and knock-in mice (Hu et al., 2009; Hu et al., 2011). The knock-in mutants include CDK6^R31C^ (*R31C*), a hyper-active, inhibitor-resistant kinase that cannot interact with the INK4 family inhibitor proteins (Pavletich, 1999), and CDK6^K43M^ (*K43M*) (Hu et al., 2011), a catalytically inactive kinase. The *R31C* mutant mimics hyperactivation of CDK6 in diseased cells, whereas the catalytic inactive *K43M* mutant mimics pharmacological inhibition of kinase activity. Employing these defined *Cdk6* mouse models, we have shown that CDK6 plays an important role in proliferation and differentiation of various precursors in addition to its canonical role as a cell cycle regulator. For instance, in the hematopoietic system, loss of CDK6 or its kinase activity in mice results in a reduction in the number of bone marrow (BM)-resident hematopoietic stem cells (HSCs) (Hu et al., 2009; Hu et al., 2011), accompanied by significantly lower levels of circulating T cells (Malumbres et al., 2004; Hu et al., 2009; Hu et al., 2011). In contrast, mice with constitutively active kinase activity of CDK6 have higher numbers of BM-resident HSCs (Hu et al., 2009; Hu et al., 2011) and T cells (Hu et al., 2011). Moreover, CDK6 is found to be essential for proliferation of the neural stem cells (NSCs) within the dentate gyrus and the sub-ventricular zone areas (Beukelaers et al., 2011). Combined with other specific effects of CDK6 such as on the production of antioxidants including reduced nicotinamide adenine dinucleotide phosphate (NADPH) and glutathione (GSH) (Wang et al., 2017), induction of angiogenesis (Kollmann et al., 2016), and acting as a downstream effector of Notch1 signaling pathway (Hu et al., 2009; Hu et al., 2011; Jena et al., 2016; Hou et al., 2018), which is involved in the maintenance of the stemness of HSCs (Palomero and Ferrando, 2008), the evidences described above suggest that CDK6 kinase activity is required for regulation of the fate of HSCs and NSCs, and that inhibition and promotion of CDK6 kinase activity (Jena et al., 2016) could be a novel therapeutic intervention in the treatment of cancers such as leukemia (Jena et al., 2016) or neurodegenerative diseases such as Alzheimer’s Diseases (AD), respectively.

In our previous studies, we have also found that mice lacking the CDK6 protein or its kinase domain (*K43M*) exhibited more beige cell formation in the subcutaneous white adipose tissues (SAT) but not in the visceral white adipose tissues (VAT) (Hou et al., 2018), greater energy expenditure (Hou et al., 2018), better glucose tolerance and higher insulin sensitivity (Hou et al., 2018), and were more resistant to high fat diet-induced obesity (Hou et al., 2018). The effect of CDK6 on white fat browning was cell autonomous since re-expression of CDK6 in *Cdk6* ^*−/−*^ mature or precursor cells reversed the phenotypes. Although the precise mechanism of regulation of adipose tissues by CDK6 is still undergoing investigation, we have shown that CDK6 negatively regulates white fat browning partially through kinase-mediated suppression of RUNX1, a transcription factor that normally promotes the expression of *Ucp-1* and *Pgc-1α* expression (Hou et al., 2018), two brown fat specific genes known to induce browning process (Velickovic et al., 2019). Moreover, we have also found that RUNX1 mediates CDK6’s effects on precursor commitment to beige cells on SAT, since ablation of RUNX1 in *K43M* adipocyte precursors reversed the defect of precursor differentiation into white adipocytes (Hou et al., 2018). However, it is unknown at present how CDK6 affects VAT biology, which is of particular importance because it is linked to metabolic dysfunction and increased risk of heart diseases and non-insulin dependent diabetes much more so than SAT, as reduction of fat mass in VAT is not through white fat browning (Hou et al., 2018).

In this study, we have identified the molecular mechanisms whereby CDK6 affects VAT metabolism by maintaining precursor numbers through promotion of stem cell proliferation and differentiation. Thus, pharmacological attempts at inhibition of CDK6 kinase activity present a novel treatment of obesity and the related metabolic diseases.

## 2 Materials and methods

### 2.1 Mice

*WT, K43M, R31C, K43M;Runx1* ^*fl/fl*^ *;CRE* ^*-*^ *, and K43M;Runx1* ^*fl/fl*^ *;CRE* ^*+*^ mice were generated as described in our previous studies (Hu et al., 2009; Hu et al., 2011; Hou et al., 2018). Equal numbers of both male and female mice were used for all the experiments. Most of the figures shown were derived from male mice. There was no significant difference between male and female. Animal experiments were approved by the Institutional Animal Care and Use Committee of Tufts University.

### 2.2 Virus

A retroviral expression vector (MigR1-GFP-CDK6) carrying the mouse *Cdk6* cDNA was constructed as previously described (Jena et al., 2016)*.* MigR1-GFP-CRE, a gift from Dr. Richard Van Etten, is a retroviral expression vector carrying the GFP-CRE cDNA (Walz et al., 2012).

### 2.3 Antibodies

Antibodies used in this study included CDK6 (C-21) from Santa Cruz), α-Tubulin from Sigma, RUNX1 from Abcam, and Sca-1-FITC/APC, CD36-APC, CD31-APC, CD45-APC, TER119-APC, CD24-FITC, CD29-FITC, CD34-FITC from e-Bioscience, BrdU monoclonal antibody (MA3-071) from ThermoFisher, eBioscience™ BrdU Staining Kit for Flow Cytometry FITC from ThermoFisher (8811-6600-42), and 7-Aminoactinomycin D (7-AAD) from ThermoFisher (A1310).

#### 2.3.1 Immunostaining

Immunostaining was done as described (Brown et al., 2012). Briefly, adipose tissues were fixed in 10% buffered formalin overnight, embedded in paraffin, and sectioned. For BrdU staining, Sections were dewaxed, rehydrated, washed in PBS, and subjected to antigen retrieval by a pressure cooker following “IHC antigen retrieval protocol” (Abcam). The retrieved sections were incubated overnight at 4°C in a humidified chamber with BrdU monoclonal antibody (ThermoFisher, MA3-071) in 2% BSA-PBS at a dilution of 1:50. Sections were washed with PBST and incubated with a secondary antibody in PBS at room temperature in the dark for 1 h. Immune complex formation was detected through immunofluorescence using Alexa Fluor 488 (green, ThermoFisher) conjugated antibodies. The nuclei were stained with DAPI (blue). Samples were also counterstained with Hematoxylin and Eosin (H&E) staining, which was performed by core facility of TUFTS Medical School. Images were taken at a magnification of ×40.

### 2.4 *In vivo* proliferation assay

Male and female 4-month-old *WT*, *K43M*, *R31C,* and *K43M;Runx1* ^*−/−*^ mice were intraperitoneally injected with saline or BrdU (Sigma Co., St. Louis, MO) at a concentration of 50 μg/g of body weight twice daily, at 7.00 a.m. and 6.00 p.m. for 3 consecutive days (total 7 injections) (Staszkiewicz et al., 2009). Animals will be euthanized 24 h after the last injection of BrdU. Label-retaining cells (LRC) will be quantified in fat depots by flow cytometric analysis, immunohistochemical methods, and immunofluorescent analysis.

### 2.5 Flow cytometry.

Four color flow cytometry was done as described (Gounari et al., 2002; Gounari et al., 2005; Hu et al., 2009; Hu et al., 2011; Jena et al., 2016). Briefly, for Lin^+^ populations, cells were stained with the combination of Lin^+^ cells including APC conjugated CD45, TER119, and CD31 of lineage-specific antibodies as described (Oguri et al., 2020), and other cell surface makers including FITC conjugated CD24, CD29, CD34, and Sca-1 (Rodeheffer et al., 2008). For Sca-1^+^ and Sca-1^+^CD36^+^ populations, cells were stained with antibodies against cell surface markers Sca-1, or Sca-1 and CD36. For Sca-1^+^BrdU^+^ staining, a standard cell surface staining with Sca-1-APC was performed following BrdU staining (FITC) using eBioscience™ BrdU Staining Kit according to manufacturer’s instruction. The stained cells were then analyzed by flow cytometry analysis (Cyan flow cytometer from Beckman Coulter). For Sca-1^+^7AAD staining, we performed a standard cell surface staining with Scal-1-FITC. Following fixation and permeabilization, cells were then stained with 7-AAD for 15 min at room temperature according to manufacturer’s instruction. For PI staining, control (CTR) and *KO* cells were seeded into a 6-well plate at a density of 2×10^5^ cells/well. After 24h, cells were trypsinized and fixed with ice-cold 70% ethanol overnight. Cells were permeabilized using PI/RNase staining buffer (550,825, BD Pharmingen, San Diego, CA, United States) and incubated for 45 min at room temperature. DNA content was determined by Cyan flow cytometer. The data was analyzed by FlowJo™ 10.8.2 software.

### 2.6 Generation of ADSCs from VAT of *WT, K43M, R31C, and K43M;Runx1* ^*fl/fl*^ *;CRE* ^*-*^ mice

ADSCs were isolated from VAT of 6-wk-old *WT*, *K43M*, *R31C,* and *K43M;Runx1* ^*fl/fl*^ *;CRE* ^*-*^ mice and grown *in vitro* (Hou et al., 2018)*.* Epididymal white adipose tissue (eWAT) from male mice and gonadal white adipose tissue (gWAT) from female mice were used in this study. ADSCs were isolated from the adipose stromal vascular fraction (SVF) according to a published procedure (Permana et al., 2004). Briefly, freshly collected eWAT and gWAT from normal chow-fed animals were digested in 1 x HBSS containing 1 mg/mL type I collagenase (Millipore Sigma, United States) at 37°C for 1 h. The suspension was filtered through a sterile 100 µm nylon mesh (Thermofisher Scientific) and centrifuged at 1,000 rpm for 5 min. The pellet fraction containing pre-adipocytes was washed 2 times with PBS +2% FBS and then incubated with red blood cell lysis buffer to remove red blood cells. Cells were then filtered through a sterile 70 µm nylon mesh (Thermofisher Scientific) and centrifuged at 1,000 rpm for 5 min and counted. Fractions of 3×10^5^ cells were used for subsequent staining and analysis using flow cytometry.

### 2.7 Generation of MEFs from *WT, K43M, and R31C* mice

MEFs were isolated from 12.5 to 13.5 days-post-coitus pregnant *WT, KO, K43M,* and *R31C* mice as previously described (Jain et al., 2014).

### 2.8 *In vitro* differentiation assay

The primary ADSCs, a. k.a. pre-adipocytes, from the SVF of e/gWAT of *WT*, *K43M, R31C,* and *K43M;Runx1* ^*fl/fl*^ *;CRE* ^*-*^ mice or the primary MEFs derived from *WT*, *K43M,* and *R31C* mice were plated in DMEM supplemented with 10% FBS, 50 IU/mL penicillin, 50 μg/mL streptomycin and 2 mM L-glutamine. After confluency was reached, ADSCs were differentiated for 7 days in differentiation medium containing 20 nM insulin, 25 nM dexamethasone, and 0.5 mM 3-Isobuyl-1-methylxanthine (IBMX). *In vitro,* confluent primary ADSCs were stimulated with white fat inducers for 7 days (Tang and Lane, 2012). Accumulation of lipid-containing cells was detected by Oil Red O staining as described (Hansen et al., 1999). In a subset of experiments, differentiated cells were also used to purify RNA or proteins for analysis of WAT markers using RT-PCR and immunoblotting.

To re-express CDK6 in *K43M* MEFs, the isolated *K43M* MEFs were transduced with *Cdk6*-IRES-GFP (CDK6-GFP) or control IRES-GFP (GFP) virus as described (Jena et al., 2016). The differentiation assay was then performed as described above.

To delete RUNX1 in *K43M* ADSCs, ADSCs isolated from eWAT/gWAT of *K43M;Runx1* ^*fl/fl*^;CRE^−^ mice or the control *K43M;RUNX1* ^*+/+*^ mice were infected with retroviral vector MigR1-GFP-CRE (Jena et al., 2016) encoding GFP-CRE. The infection efficacy was determined to be approximately 90%, at a similar level as described in previous studies (Reschly et al., 2006; Jena et al., 2016; Hou et al., 2018).

### 2.9 Immunoblotting and IP-Western

Cell lysates were prepared as described (Hu et al., 2009). Western blotting was performed as described (Hu et al., 2009). Antibodies used in the studies including CDK6 from Santa Cruz, RUNX1 from Abcam, Vinculin from Cell Signaling (Cat. 4,650), and α-Tubulin from Sigma.

### 2.10 RT-PCR

The experimental procedures were the same as those described previously (Zhang et al., 2012). The *36B4* gene, encoding an acidic ribosomal phosphoprotein P0 (RPLP0), was used as internal control. The primer sequences for WAT-associated transcriptional factors including PPARα, PPARγ, C/EBPα, and C/EBPβ and WAT related genes including *Fabp4, Adiponectin (AdipoQ),* and *Leptin* were listed in the Supplementary Table S1.

### 2.11 CRISPR/Cas9-mediated knockout of Cdk6 gene in 3T3-L1 cells

The experimental procedures were the same as described (Zhang et al., 2022). Target CDK6 gene specific guide RNAs were designed (http://crispr.mit.edu) and cloned into lentiCRISPR v2 vector with puromycin resistance (Cat. 52961, Addgene, Watertown, MA, United States). Cells were selected by puromycin (1 ug/mL) for 2 weeks, followed by single colony picking and expansion. CDK6 knockout efficiency of individual clones were validated by Western blotting. The sequence of guide RNAs for mouse are CDK6-mus-1# 5′-CGG​CGA​AGG​CGC​CTA​TGG​GA-3′, CDK6-mus-2# 5′-CTA​GGC​CAG​TCT​TCC​TCT​CC-3’.

### 2.12 Statistical methods and statistical analysis

For most experiments, the sample size was chosen based on expected differences between experimental and control groups to provide adequate power to detect a significant difference specifying α = 0.05, two-tailed testing, and power (1-β) of 80%, using commercially available software packages (Statistical Solutions nQuery Advisor; http://www.statsol.ie/nquery/nquery.htm). All data were expressed as means ± SE. We calculated statistical significance using Student’s t-test or one way ANOVA, with *p < 0.05 being considered significant.

## 3 Results

### 3.1 CDK6 kinase activity induces obesity

**FIGURE 1:**
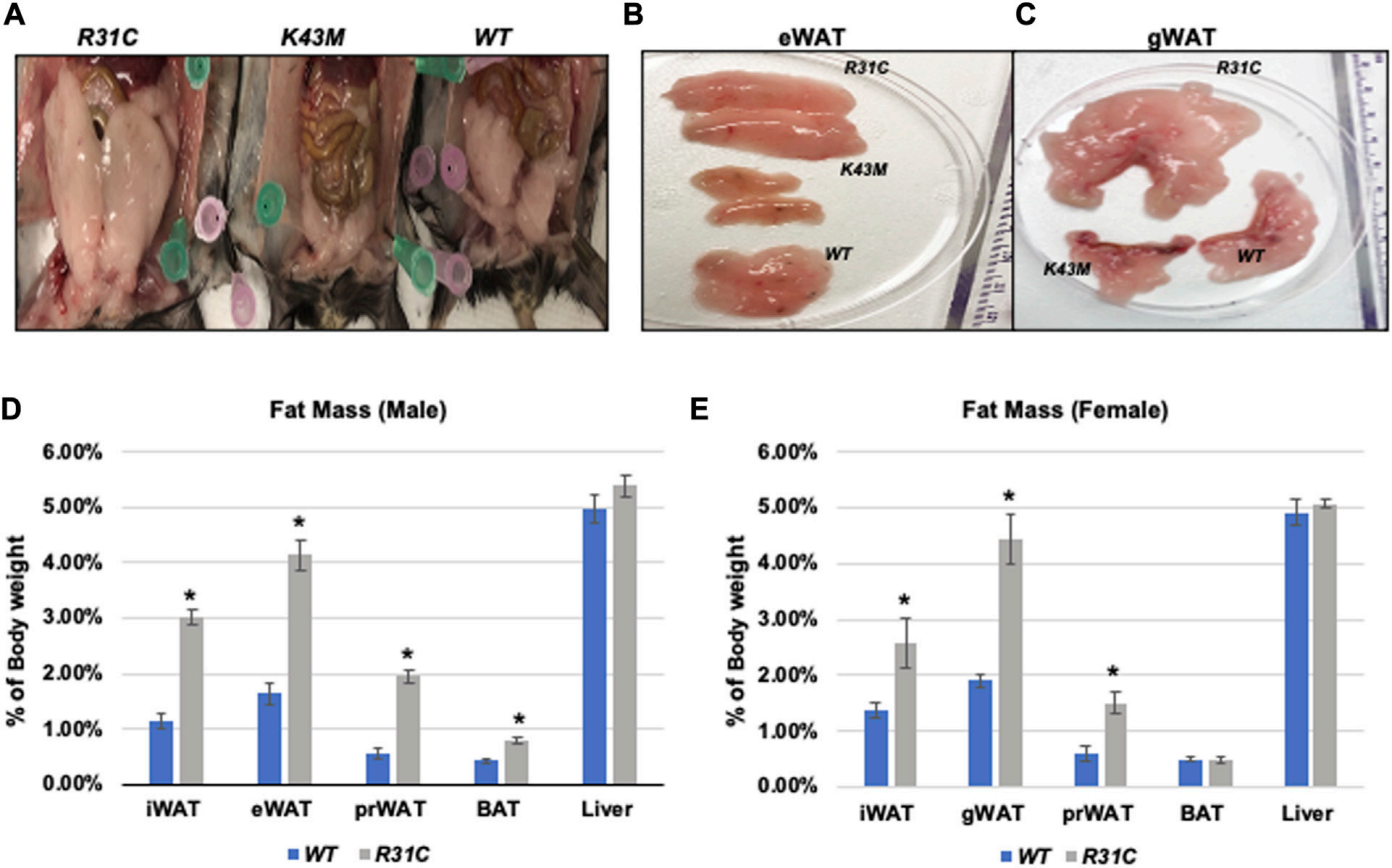
*Reduced fat mass in K43M mice and increased fat mass in R31C mice.* **(A)** Ventral view of female *WT, K43M,* and *R31C* mice fed with NCD. Appearance of a close view of the eWAT **(B)** and gWAT **(C)** from the mice of the three genotypes. **(D)** Mass of various fat pads was normalized to body weight of male mice on NCD at 18–20 weeks of age. **(E)** Mass of various fat pads was normalized to body weight of female mice on NCD at 18–20 weeks of age. For **(D, E)**, data shown are mean ± SE (*n* = 6 for each group), *p < 0.05, t-test, vs. *WT* of the corresponding adipocyte tissue.

We have previously shown that mice lacking the CDK6 kinase domain (K43M) exhibited metabolic reprogramming (Hou et al., 2018) as manifested by increased white fat browning, reduced fat mass in both SAT and VAT, more resistance to high fat diet (HFD)-induced obesity, improved metabolic profiles, and enhanced insulin sensitivity (Hou et al., 2018). To further understand the role of CDK6, especially its kinase activity, in obesity, we examined fat compositions in the constitutively active CDK6 mutant (*R31C*) mice and found that *R31C* mice had significantly increased fat pad mass including iWAT (3-fold for male, 1.87-fold for female), eWAT (2.52-fold for male, 2.33-fold for female), peri-renal WAT (prWAT, 3.53-fold for male, 2.52-fold for female), brown adipose tissue (BAT, 1.85-fold for female, but no significant difference observed in male) (Figures 1A–E), as compared with *WT.* By contrast, liver weight was not significantly different between *WT* and *R31C* mice (Figures 1D, E). These results indicate that expression of a constitutively active CDK6 enhances fat deposition. Together with our previous findings that mice carrying a kinase inactive CDK6 mutant allele (*K43M*) had a reduced fat deposition and leaner body mass, these results demonstrate that CDK6 kinase activity induces obesity.

### 3.2 CDK6 regulates the number of adipocyte precursors

**FIGURE 2:**
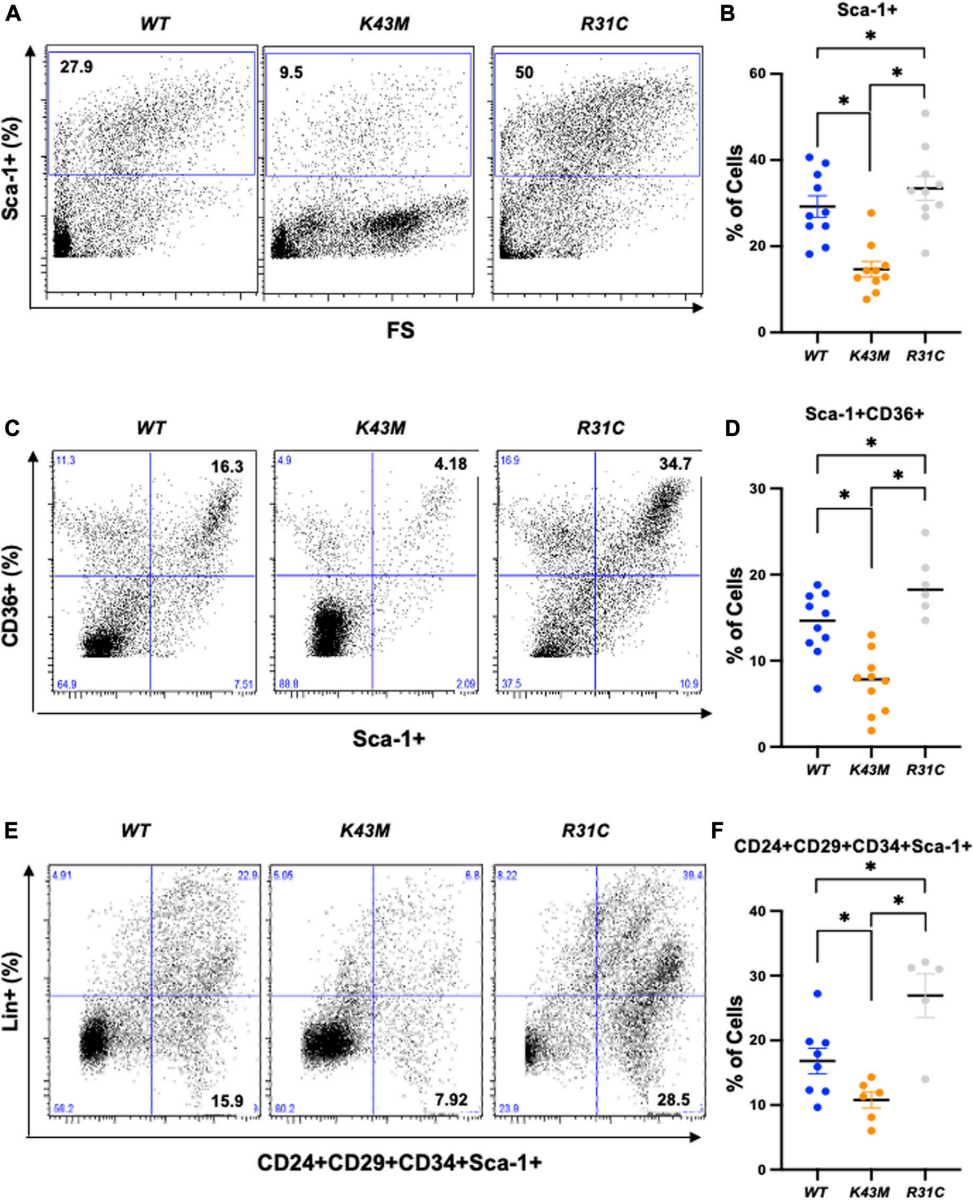
*Reduced adipocyte precursors in K43M mice and increased adipocyte precursors in R31C mice.* **(A, C, E)** Representative flow cytometric profiles of Sca-1^+^ **(A)**, Sca-1^+^CD36^+^ **(C),** and Lin^−^Sca-1^+^CD24^+^CD29^+^CD34^+^ **(E)** cells isolated from eWAT of *WT*, *K43M, and R31C* mice at 18–20 weeks of age. **(B, D, F)** Histograms summarizing the Sca-1^+^ **(B)**, Sca-1^+^CD36^+^ **(D)**, and Lin^−^Sca-1^+^CD24^+^CD29^+^CD34^+^ **(F)** cells in panel **(A, C, E)**, respectively. For **(B, D, F)**, data shown are mean ± SE (*n* = 6–10). *p < 0.05, t-test. For B, *p*-value < 0.0001, one way ANOVA, for D, *p*-value < 0.0001, one way ANOVA, and for F, *p*-value = 0.0008, one way ANOVA.

First, we examined if reduced VAT mass in *K43M* mice, a phenomenon that was reported previously (Hou et al., 2018), and elevated VAT mass in *R31C* mice (Figure 1) was caused by a change in the number of adipocyte precursors including stem cells and progenitors. Adult SVF of fat depots contain ADSCs, a population of somatic stem cells that can renew themselves as well as differentiate into adipocytes (Zuk et al., 2001; Gimble and Guilak, 2003). We determined the number of precursors by analyzing the expression of Sca-1^+^, Sca-1^+^CD36^+^, and Lin^−^Sca-1^+^CD24^+^CD29^+^CD34^+^ cells in the SVF using flow cytometry. We found that male *K43M* SVF of eWAT had a 50, 48, and 36% reduction, respectively, in Sca-1^+^, Sca-1^+^CD36^+^, and Lin-Sca-1^+^CD24^+^CD29^+^CD34^+^ cells, as compared to *WT* SVFs (Figure 2). In contrast, male *R31C* SVFs had a 1.14-, 1.33-, and 1.60-fold increase, respectively, in the three stem and progenitor cell populations, as compared to those of *WT* SVFs (Figure 2). Similarly, female *K43M* SVF of eWAT (Supplementary Figure S1) had a 45, 50, and 36% reduction, respectively, in Sca-1^+^, Sca-1^+^CD36^+^, and Lin-Sca-1^+^CD24^+^CD29^+^CD34^+^ cells, as compared to *WT* SVFs. In contrast, female *R31C* SVFs had a 1.6-, 1.2-, and 1.6-fold increase, respectively, in the three stem and progenitor cell populations, as compared to those of *WT* SVFs (Supplementary Figure S1). Thus, the kinase activity of CDK6 likely governs stem cell/progenitor numbers in the e/gWAT.

### 3.3 CDK6 regulates proliferation of adipocyte precursors

To understand if decreased fat mass in *K43M* mice and increased fat mass in *R31C* mice are attributed to the changes of proliferation status of adipocyte precursors, we performed bromodeoxyuridine (BrdU) incorporation experiments. BrdU is a synthetic analogue of thymidine that can be incorporated into cells during DNA replication, which allows successful identification and quantification of the dividing cells in different fat depots by immunofluorescent and flow cytometric methods. It has been shown that BrdU-positive ADSCs are also positive for Sca-1^+^ but negative for the hematopoietic lineage (CD45^-^/CD4^-^) (Staszkiewicz et al., 2009).

**FIGURE 3:**
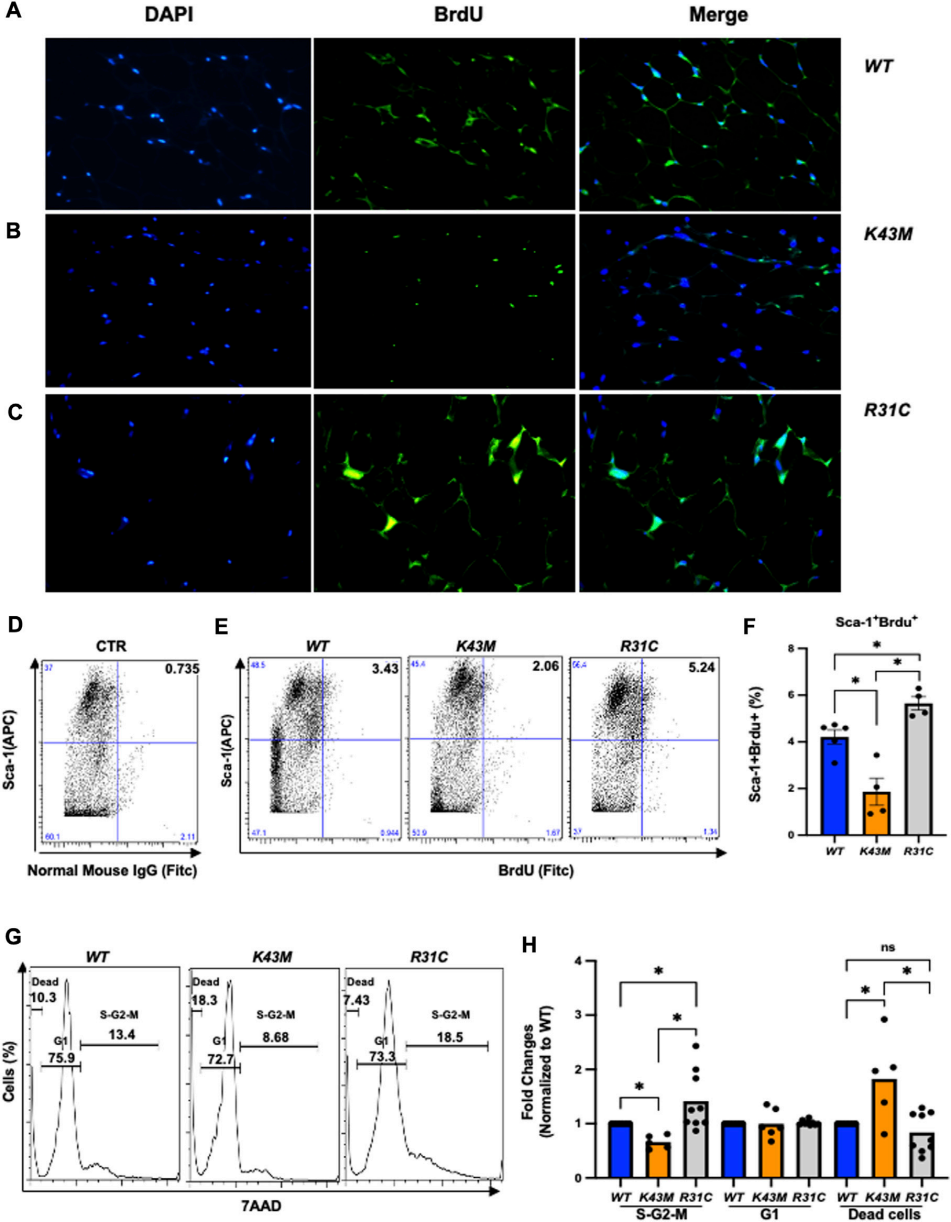
*Role of CDK6 kinase activity in BrdU incorporation and cell cycle profiles.* **(A-C)** Representative immunofluorescent detection of BrdU-labeled cells (green) in eWAT derived from 4-month-old male *WT*, *K43M, and R31C* (*n* = 4) mice which were administered BrdU for three consecutive days. Cell nuclei counterstained with DAPI (blue). Magnification ×40. **(D, E)** Representative flow cytometric profiles of negative control with SVF cells stained with normal mouse IgG-FITC and Sca-1-APC **(D)** and Sca-1^+^BrdU^+^ cells isolated from eWAT of male *WT*, *K43M, and R31C* mice at 18–20 weeks of age **(E)**. **(F)** Histograms summarizing the Sca-1^+^BrdU^+^ cells in panel **(E)**. **(G)** Representative flow cytometric profile profiles of Sca-1^+^7AAD^+^ cells isolated from eWAT of male *WT*, *K43M, and R31C* mice at 18–20 weeks of age. **(H)** Histograms summarizing the Sca-1^+^7AAD^+^ cells in panel **G**. For **F,** data shown are mean ± SE (*n* = 4–5), *p < 0.05, t-test. For **H**, data shown are fold change of cells in different cell cycle phases normalized to the relative *WT* controls, which was arbitrarily defined as 1 unit. Data shown are mean ± SE (*n* = 5–9). *p < 0.05, t-test. For 3F, *p*-value = 0.0002, one way ANOVA, and for 3H, *p*-value of S-G2-M = 0.0027, *p*-value of G1 = 0.9521, and *p*-value of dead cells = 0.0013.

BrdU-labeled cells were detected and quantified in fat deports by a standard immunofluorescent in conjunction with multiple fluorescently labeled cell surface markers, allowing extensive phenotypic characterization of dividing cells in addition to assessment of their frequency (Figure 3; Supplementary Figure S2). Consistent with our previous studies (Hou et al., 2018), we found that *K43M* eWAT had smaller size of cell body compared with *WT* eWAT, while R31C eWAT have comparable cell size as *WT* eWAT (Supplementary Figure S2A-C). To confirm BrdU was incorporated into the nucleus, we performed double-immunofluorescence staining using anti-BrdU antibodies together with DAPI staining (Figures 3A–C, DAPI and BrdU). As expected, BrdU staining co-localized with DAPI stained nuclei (Figures 3A–C, merge). *WT* eWAT had 62.56% ± 4.00%, while *K43M* eWAT and *R31C* eWAT had 38.56% ± 3.84%, and 75.10% ± 1.76% double positive cells (Supplementary Figure S2D)., respectively, significantly different from *WT* eWAT, indicating that loss of CDK6 kinase activity in precursors reduced proliferation of cells, while gain of function of CDK6 kinase activity in precursors increased proliferation of cells.

To accurately quantify BrdU incorporation, a standard cell surface staining with Scal-1-APC was performed following BrdU staining (FITC) by flow cytometry analysis. SVF of the eWAT from *K43M* mice displayed a 55% (male) (Figures 3D–F) and a 42% (female) (Supplementary Figure S3A-C) reduction in Sca-1^+^BrdU^+^ cells as compared to that from *WT* mice. In contrast, *R31C* SVF of the eWAT had a ∼1.3-fold for both male and female (Figures 3D–F; Supplementary Figure S3A-C) increase, respectively, in Sca-1^+^BrdU^+^ cells, as compared with *WT* SVF of the eWAT. These data indicate that loss of CDK6 kinase activity resulted in a reduction of BrdU incorporation into ADSCs, while constitutively active CDK6 kinase activity increases BrdU incorporation of ADSCs, further demonstrating a role of CDK6 in the proliferation of adipocyte precursors.

To further confirm the proliferative status of ADSCs, we performed a standard cell surface staining with Scal-1-FITC followed by 7AAD (PE-cy5) staining. Flow cytometric analysis revealed that *K43M*-Sca-1^+^ cells had a 34% (male) and a 28% reduction in the S/G2/M phase, respectively, and a 1.8- fold (male) and a 1.5-fold (female) increase, respectively, in the dead cells (Figures 3G, H; Supplementary Figure S3D) relative to that of *WT* mice. In contrast, *R31C-*Sca-1^+^ had a 1.4- (male), and 1.6-fold (female) increase, respectively, in the S/G2/M phase, and a 16% (male) and a 9% (female) reduction in dead cells, respectively, as compared with *WT* (Figures 3G,H; Supplementary Figure S3D, E). These results indicate that loss of CDK6 kinase activity decreased proliferation of Sca-1^+^ ADSCs and increased cell death, while constitutively active CDK6 kinase activity increased proliferation and decreased cell death, suggesting that CDK6 kinase activity is required for stem cell proliferation and survival.

### 3.4 Re-expression of CDK6 in K43M-MEFs rescued the defect of K43M in differentiation

The reduced VAT in *K43M* mice (Hou et al., 2018) could also be due to the defect of *K43M* precursors to differentiate into adipocytes. To examine this possibility, we chose precursors isolated from both MEFs and SVFs to study the effect of CDK6 on differentiation. ADSCs (Zuk et al., 2001; Gimble and Guilak, 2003) shares a number of similarities, although not identical, to bone marrow derived mesenchymal stem cells (BMSCs), for instance, they both contain large population of stem cells with multi-lineage differentiation capacity (Bunnell et al., 2008). MEFs have been utilized as a surrogate stem cell model for BMSCs to study mesoderm-type cell differentiation such as adipocytes (Saeed et al., 2012) due to the characteristics of easy accessibility, simplicity of isolation, self-renewal and proliferation ability, multilineage differentiation potentials, and immunomodulatory effects (Hansen et al., 2004; Abella et al., 2005; Saeed et al., 2012).

**FIGURE 4:**
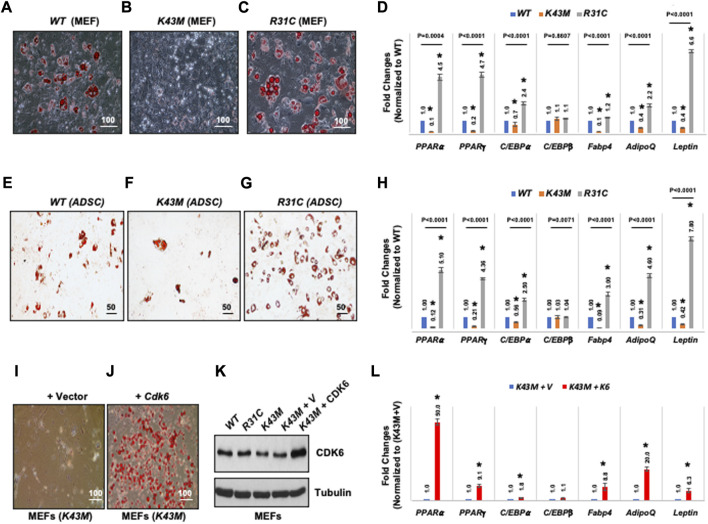
CDK6 kinase activity is required for differentiation of precursors derived from MEFs and ADSCs into adipocytes. **(A–C, E–G)** Representative images of Oil Red O staining of differentiated cells from MEFs **(A–C)** or SVFs **(E–G)** isolated from *WT*, *K43M*, and *R31C* mice in the presence of WAT inducers. **(D, H, L)** qRT-PCR analyses for expression levels of WAT-associated transcriptional factors and WAT-related genes with 36B4 mRNA as an internal control. Data shown are fold changes of each mRNA normalized to the relative controls, either WT **(D, H)** or (*K43M* + V) **(L)**, which was arbitrarily defined as 1 unit. **(I, J)** *K43M* MEFs transduced with MigR1-Vctor [**(I)**, *K43M* + V] and with MigR1-*Cdk6* [**(J)**, *K43M* + *Cdk6*] were grown to confluent and then induced to differentiate. At day 8 post-induction, cells were stained for lipid droplets with Oil Red O. **(K)**, Immunoblots of the protein levels of CDK6 from the differentiated cells **(I, J)**. Tubulin was used as loading control. For **(D, H, L)**, data shown are mean ± SE (*n* = 3). *, *p* < 0.05, T-test, significantly different from the respective control. For 4D and 4H, p value was labeled on the top of each group, one way ANOVA.

To determine if CDK6 participates directly in specification or differentiation of adipocytes from an earlier precursor (Shao and Lazar, 1997; Hansen et al., 1999; Liu et al., 2007), we compared the capacity of MEFs derived from *WT, K43M* and *R31C* mice to differentiate into adipocytes *in vitro* in response to adipogenic factors. As shown in Figure 4, *WT* MEFs could differentiate as a mixed population into lipid-containing cells as assayed via Oil Red O staining (Figure 4A) with significantly higher level of mRNAs including WAT associated transcriptional factors including (PPARα, PPARγ, C/EBPα) and WAT related genes including *Fabp4, Adiponectin (AdipoQ)* and *Leptin* than *K43M* MEFs (Figure 4D). Similar results were obtained in analyses of ADSCs isolated from the SVFs of *WT and K43M* mice (Figures 4E–H). In contrast, *R31C* MEFs and *R31C* ADSCs had much more Oil Red O-positive cells (Figures 4C,G) and significantly increased mRNA levels of transcriptional factors and WAT related genes (Figures 4D,H) than *WT* MEFs and ADSCs. Of note, the mRNA level of *C/EBPβ* did not change among three different cells. Thus, adipocyte precursors with no CDK6 kinase activity failed to differentiate into white adipocytes, while those with constitutively active CDK6 kinase activity had enhanced ability to differentiate into white adipocytes.

To confirm that the above phenotypes were due to a cell-autonomous effect of CDK6, we sought to determine if re-expression of CDK6 in *K43M* MEFs *in vitro* could promote differentiation of *K43M* MEFs towards white adipocytes. To re-express CDK6 in *K43M* cells, MEFs were isolated and transduced with the control IRES-GFP (*K43M + V,* Figure 4I) vectors or the *Cdk6*-IRES-GFP (*K43M + Cdk6,* Figure 4J) as described (Jena et al., 2016). The differentiation assay was then performed as described above. Re-expression of CDK6 in *K43M* MEFs (Figure 4K) reversed the defect of *K43M* cells in WAT differentiation (Figure 4I-L), as manifested as increased Oil Red O-positive cells (Figure 4J) and significantly increased mRNA levels of WAT-associated transcriptional factors and WAT-related genes (Figure 4L) than *K43M* MEFs transduced with vectors (Figure 4L). These data suggest that the absence of CDK6 kinase activity restricts adipocyte precursors from executing WAT adipogenic programs in a cell-autonomous manner, and that CDK6 is required for WAT vs*.* BAT lineage commitment.

### 3.5 Knock-down of CDK6 in 3T3-L1 cells copied the defect of K43M in differentiation

**FIGURE 5:**
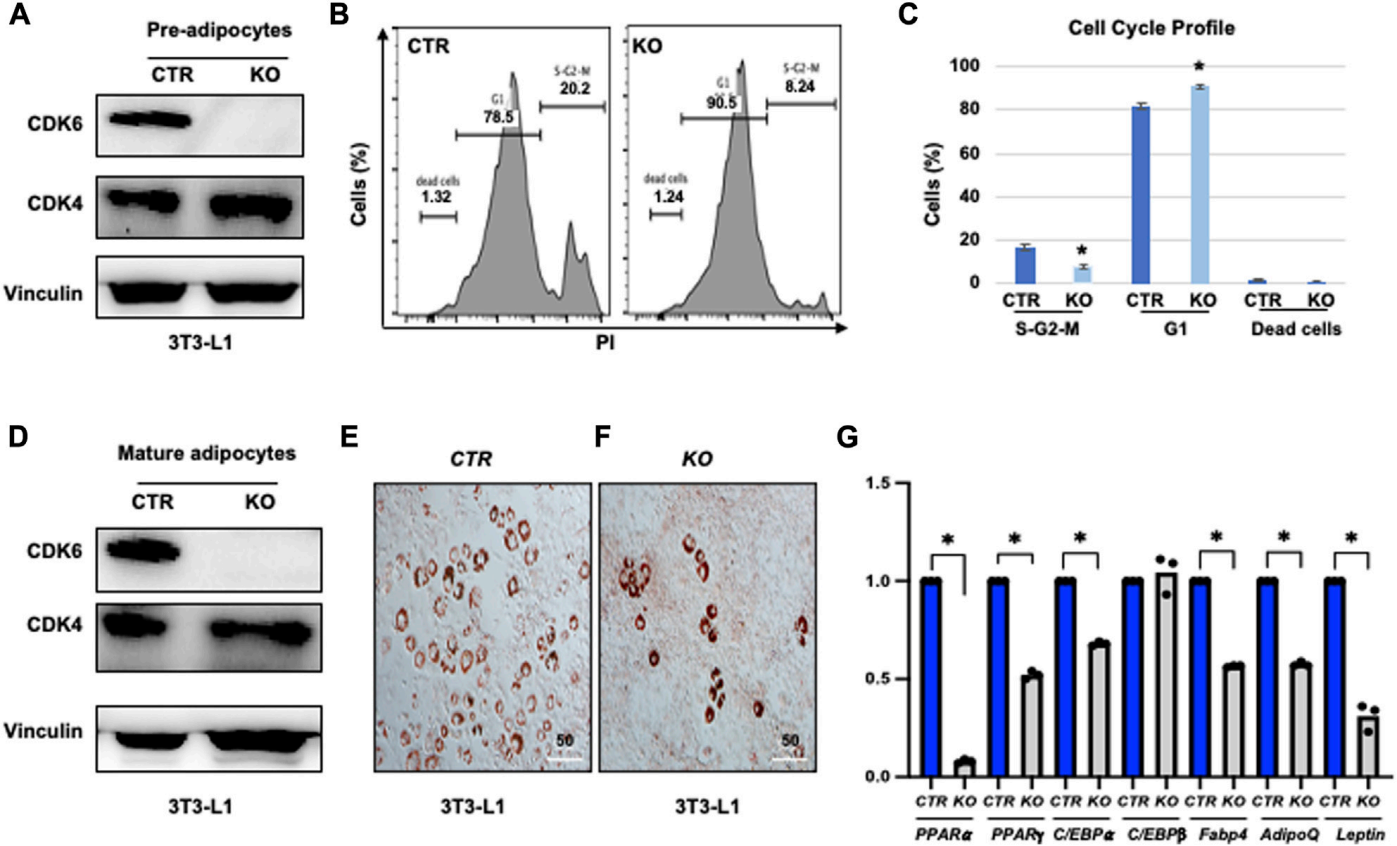
Knock-down of CDK6 in 3T3-L1 cells copied the defect of *K43M* in differentiation. **(A, D)** Immunoblots of the protein levels of CDK6 and CDK4 in pre-adipocytes **(A)** and differentiated cells **(D)** of control (CTR) and *KO* cells. Vinculin was used as loading control. **(B)** Representative flow cytometric cell cycle profiles of PI+ pre-adipocytes. **(C)** Histograms summarizing the cell cycle distribution of the cells in **(B)**. Data shown are mean ± s.e. (*n* = 3); *p <0.05 vs CTR, T-test. **(E, F)** Representative images of Oil Red O staining of differentiated cells from CTR **(E)** or *KO* **(F)** cells in the presence of WAT inducers for 7 days. **(G)** qRT-PCR analyses for expression levels of WAT-associated transcriptional factor and WAT-related genes. Data shown are mean ± SE (*n* = 3). *, *p* < 0.05, T-test, significantly different from the CTR.

To further validate the cell-autonomous effect of CDK6 in proliferation and differentiation, we ablated CDK6 gene by using Clustered Regularly Interspaced Short Palindromic Repeats (CRISPR) single-guide RNAs (sgRNAs), which completely removed the CDK6 expression in our previous studies (Zhang et al., 2022). We found that loss of CDK6 but not CDK4 (Figure 5A) in preadipocytes resulted in an approximately 2.5-fold reduction in the proliferative fractions (S-G2-M phase) and 15% increase in G1 phase in *KO* cells as compared to control cells (CTR) (Figures 5B, C). In the presence of WAT-inducers, *KO* (Figure 5D) cells had defects in differentiation, as manifested by reduced Oil Red O-positive cells (Figures 5E, F) and significantly reduced mRNA levels of WAT-associated transcriptional factors and WAT-related genes (Figure 5G) than *CTR* cells. Similar as in MEFs and ADSCs, the mRNA level of C/EBPβ did not change in *KO* or in *CTR* cells. Together, those data provide evidence that CDK6 affects VAT metabolism is due to cell-autonomous effect of CDK6 on fat precursor proliferation and differentiation.

### 3.6 Ablation of RUNX1 restored appropriate numbers of adipocyte precursors in K43M mice

Our previous studies have shown that genetic deletion of *Runx1* in *K43M* mice rescued most of the phenotype observed in *K43M* mice *in vivo* (Hou et al., 2018) including fat mass in VAT, demonstrating a downstream role for RUNX1 in CDK6-mediated effects on adipocyte biology. To investigate if RUNX1 mediates the effect of CDK6 kinase activity on the numbers of adipocyte precursors, we examined the expression of precursor markers using flow cytometry and found that male *K43M* ADSCs (eWAT) had a 42, 48, and 35% reduction, respectively, in Sca-1^+^, Sca-1^+^CD36^+^, and Lin^−^Sca-1^+^CD24^+^CD29^+^CD34^+^ cells, representing a significant reduction of the adipocyte precursors in the SFVs of *K43M* mice as compared to that of *WT* (Figure 5). In contrast, *K43M;Runx1* ^*−/−*^ (*KR*) SVFs had a 1.3-, 1.0-, and 1.1-fold increase in Sca-1^+^, Sca-1^+^CD36^+^, and Lin^−^Sca-1^+^CD24^+^CD29^+^CD34^+^ cells, as compared to *WT* SVFs (Figures 5A–F). No significant significance was found between *WT* and *KR* cells. Similarly, female *K43M* ADSCs (eWAT) had a 44, 51, and 43% reduction, respectively, in Sca-1^+^, Sca-1^+^CD36^+^, and Lin^−^Sca-1^+^CD24^+^CD29^+^CD34^+^ cells, representing a significant reduction of the adipocyte precursors in the SFVs of *K43M* mice as compared to that of *WT* (Supplementary Figure S4). In contrast, female *K43M;Runx1* ^*−/−*^ (*KR*) SVFs had similar populations in Sca-1^+^, Sca-1^+^CD36^+^, and Lin^−^Sca-1^+^CD24^+^CD29^+^CD34^+^ cells, as compared to *WT* SVFs (Supplementary Figure S4A-F), but significantly increased in all three populations, as compared to *K43M* SVFs (Supplementary Figure S4A-F). Thus, ablation of *Runx1* rescued the defect of *K43M* mice in the production of adipocyte precursors. The effect of RUNX1 is non-cell autonomous, since it is known that Adiponectin-CRE (*Adipoq-CRE*) mice that were used in this study express CRE in the mature adipocyte (Cristancho and Lazar, 2011) within WAT and BAT but not in the stem cell compartments (Cristancho and Lazar, 2011).

### 3.7 Ablation of RUNX1 rescued the defect of K43M on proliferation and differentiation of ADSCs

To investigate if RUNX1 mediated the effect of CDK6 kinase activity on proliferation of adipocyte precursors, we injected 4∼5-month-old *WT*, *K43M*, and *KR* mice intraperitoneally with BrdU (50 mg/kg twice daily for 3 days) and examined the proliferation status of adipocyte precursors by immunofluorescence. Consistent with Figure 3, we found that *K43M* eWAT had smaller size of cell body compared with *WT* eWAT. In contrast, *KR* eWAT have comparable cell size as *WT* eWAT (Supplementary Figure S5A-C). Similar as in Figure 3, *WT* eWAT had 64.75% ± 2.49% double positive cells, while *K43M* eWAT had 33.33% ± 4.50% double positive cells, significantly reduction compared with *WT* eWAT (Supplementary Figure S5D **)**. In contrast, *KR* eWAT had 63.65% ± 6.19% double positive cells, which is significantly increased compared with *K43M* eWAT but comparable with WT eWAT, indicating that loss of *Runx1* in mature adipocytes on *K43M* background rescued the defect of proliferation of *K43M* precursors.

To accurately quantify BrdU incorporation, a standard cell surface staining with Scal-1-APC was followed by BrdU staining (Fitc) and flow cytometry analysis. We reveled that *K43M* SVF displayed ∼40% (male) (Figures 7A–C), ∼60% (female) (Supplementary Figure S6A-C) reduction on Sca-1^+^BrdU^+^ cells, respectively, compared with *WT* SVF, which is significantly different from *WT*-SVF, while *KR ADSCs* of eWAT had a very similar populations in Sca-1^+^BrdU^+^ cells in both male and female as compared with the *WT* (Figures 7A–C; Supplementary Figure S6A-C), which is significantly different from that of *K43M* SVF, but not *WT* SVF. Therefore, loss of CDK6 kinase activity resulted in reduced BrdU incorporation of ADSC. Ablation of RUNX1 rescued the defect of K43M on BrdU incorporation of ADSC. There is no difference effect of CDK6 on proliferation of ADSC between male and female.

**FIGURE 6:**
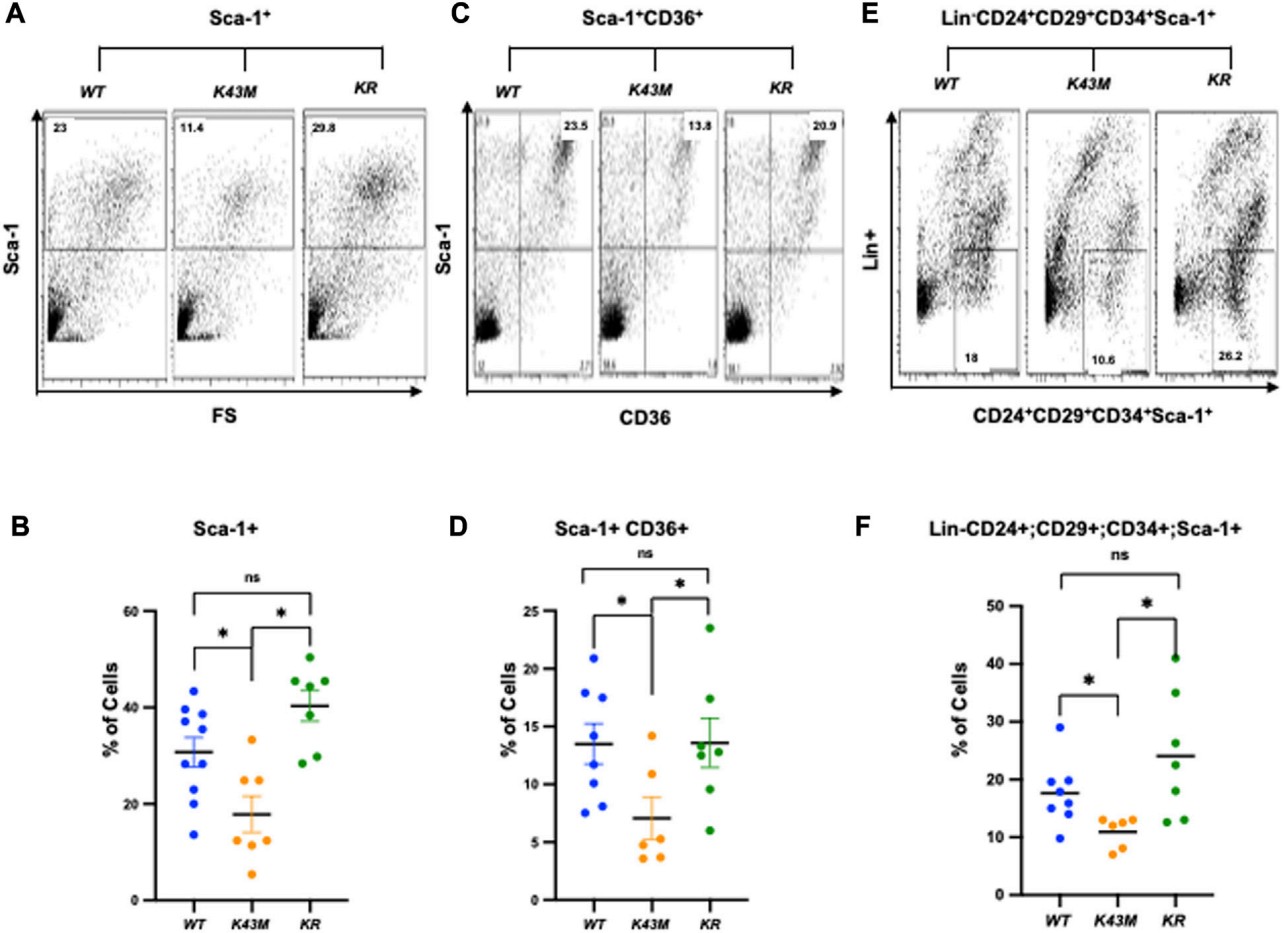
*Ablation of RUNX1 in mature adipocytes rescued the defect of precursor numbers in K43M mice.* **(A,C, E)** Representative flow cytometric profiles of Sca-1^+^ **(A)**, Sca-1^+^CD36^+^ **(C)**, and Lin^−^Sca-1^+^CD24^+^CD29^+^CD34^+^ **(E)** cells isolated from eWAT of *WT*, *K43M, and KR* mice at 4–5 months of age. **(B, D, F)** Histograms summarizing Sca-1^+^ cells in panel **(A, B),** Sca-1^+^CD36^+^ cells in panel **(C, D)**, and Lin^−^Sca-1^+^CD24^+^CD29^+^CD34^+^ cells in panel **(E, F)**. For **(B, D, F)**, data shown are mean ± SE (*n* = 6–9). *p < 0.05, t-test. For B, *p*-value = 0.0009, one way ANOVA, for D, *p*-value = 0.0406, one way ANOVA, and for F, *p*-value = 0.0164, one way ANOVA.

**FIGURE 7:**
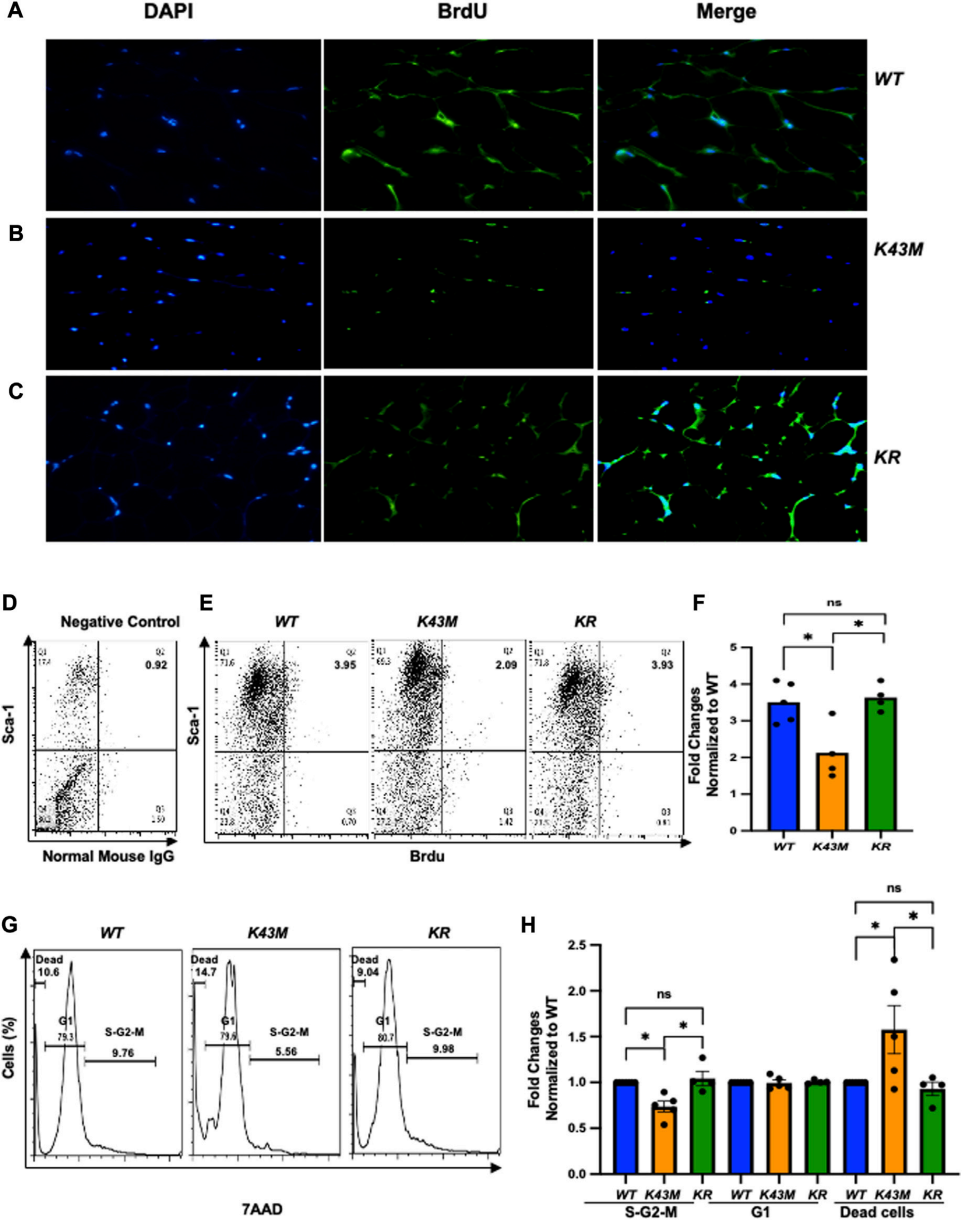
*Ablation of RUNX1 in K43M mature adipocytes rescued the defects in reduced BrdU incorporation, reduced proliferation, and increased dead cells in K43M mice.* **(A-C)** Representative immunofluorescent detection of BrdU-labeled cells (green) in eWAT derived from 4-month-old male *WT*, *K43M, and KR* (*n* = 4) mice which were administered BrdU for three consecutive days. Cell nuclei counterstained with DAPI (blue). Magnification ×40. **(D, E)** Representative flow cytometric profiles of negative control with SVF cells stained with normal mouse IgG-FITC and Sca-1-APC **(D)** and Sca-1^+^BrdU^+^ cells isolated from eWAT of male *WT*, *K43M, and KR* mice at 4–5 months of age **(E)**. **(F)** Histograms summarizing the Sca-1^+^BrdU^+^ cells in panel **(E)**. **(G)** Representative flow cytometric cell cycle profiles of Sca-1^+^7AAD^+^ cells isolated from eWAT of male *WT*, *K43M, and KR* mice at 4–5 months of age. **(H)** Histograms summarizing the Sca-1^+^7AAD^+^ cells in panel **(G)**. For **(F, H)**, data shown are fold change of Sca-1^+^BrdU^+^cells **(E)** or Sca-1^+^7AAD^+^ **(G)** normalized to the relative *WT* controls, which was arbitrarily defined as 1 unit. Data shown are mean ± SE (*n* = 4–5). *p < 0.05, t-test. For 7F, *p*-value = 0.0065, one way ANOVA, and for 7H, *p*-value of S-G2-M = 0.0010, *p*-value of G1 = 0.8963, and *p*-value of dead cells = 0.0170.

We also performed a standard cell surface staining with Scal-1-FITC followed by 7AAD (PE-cy5) and found that there was a reduction in S/G2/M phase (26% for male and 31% for female, respectively) and a 1.6-fold (male) and 1.38-fold (female) (Figures 7G,H; Supplementary Figure S6D, F) increase in dead cells in *K43M* Sca1^+^ cells (Figures 6D, E) relative to that of *WT*. In contrast, ablation of *Runx1* on *K43M* background reversed the phenotypes observed in *K43M* mice (Figures 7G,H; Supplementary Figure S6D, E). *KR* Sca-1^+^ had a comparable cell cycle profiles as *WT* Sca-1^+^, These results indicate that ablation of *Runx1* on the *K43M* background reversed the defect of *K43M* Sca-1^+^ cells in adipocyte precursor proliferation.

Taken together, these results demonstrate that CDK6 kinase activity is required for stem cell proliferation and survival and that RUNX1 is one of the major mediators downstream of CDK6 in these processes acting in a non-cell autonomous manner.

### 3.8 Ablation of RUNX1 rescued the defect of K43M on differentiation of ADSCs

**FIGURE 8:**
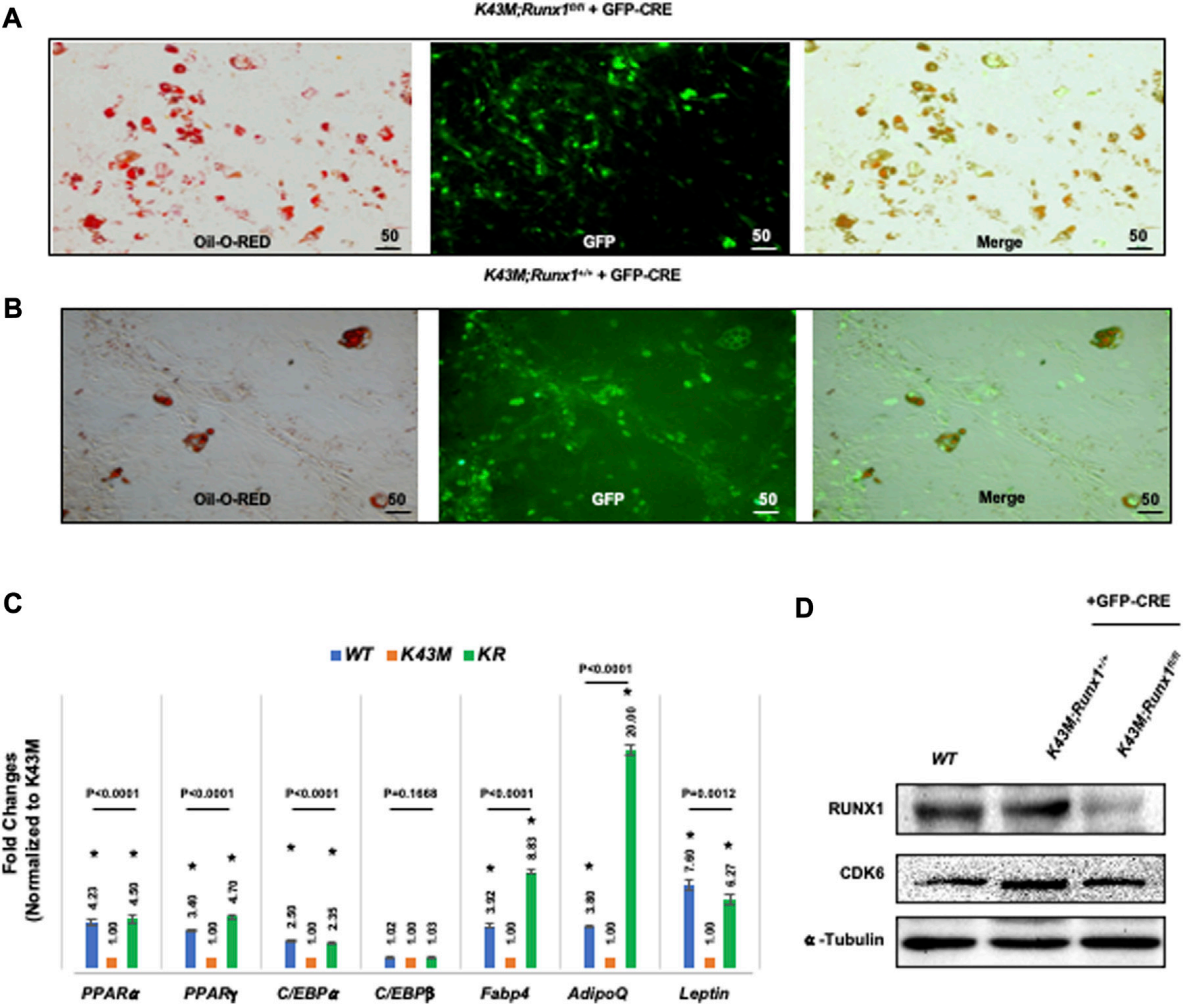
*Ablation of RUNX1 in K43M precursors rescued the defects of K43M in differentiation into white adipocytes.* **(A, B)** Fluorescent photomicrographs of differentiated cells from *K43M;Runx1* ^*fl/fl*^ + GFP-CRE **(A)** or *K43M;Runx1* ^*+/+*^ + GFP-CRE **(B)** in the presence of WAT inducers. Red fluorescence indicates positive Oil Red O staining. Green fluorescence indicates the expression of GFP-CRE. The yellow fluorescence indicates merged images from red and green fluorescence. Scale bar: 50 μm. **(C)** Relative mRNA levels of WAT related transcriptional factors and WAT markers in differentiated cells in the presence of WAT inducers. Data shown are fold change of different mutants normalized to their relative *K43M* controls, which was arbitrarily defined as 1 unit, *p < 0.05, vs*. K43M*, t-test (*n* = 5). **(D)** Immunoblots of the protein levels of RUNX1 and CDK6 in differentiated cells from 100 μg of cell lysates after 7 days in the presence of WAT inducers. α-tubulin was used as loading control. For 8c, *p*-value was labeled on the top of each group, one way ANOVA.

Next, we investigated if RUNX1 mediates CDK6’s effects on the ability of adipocyte precursors to differentiate. For deletion of *RUNX1 in vitro, K43M;Runx1* ^*fl/fl*^ and *K43M* ADSCs were infected with retroviral vector MigR1-GFP-CRE that encodes GFP-CRE (Jena et al., 2016). When these virus-transduced ADSCs reached confluency, they were stimulated with white fat inducers (Tang and Lane, 2012) for 7 days. After retroviral infection with MigR1-GFP-CRE, *K43M;Runx1* ^*−/−*^ (*K43M;Runx1* ^*fl/fl*^ + GFP-CRE) (Figure 8A ) exhibited increased WAT differentiation as compared with *K43M* (*K43M;Runx1* ^*+/+*^ + GFP-CRE) (Figure 8B), as indicated by increased Oil Red O staining (Figures 8A, B) and significantly increased WAT-associated transcriptional factors including (PPARα, PPARγ, C/EBPα) and WAT related genes including *Fabp4, Adiponectin (AdipoQ)* and *Leptin* than *K43M* cells (Figure 8C). The reduction of RUNX1 protein in the cells was confirmed by Western blotting (Figure 8D). Of note, upon deletion of Runx1 in K43M cells, the expression of *Ap2* and *Adipoq* was dramatically increased over and above that of *WT*, suggesting that either a synergistic effect of CDK6 and RUNX1 or the involvement of other molecules and mechanisms in the CDK6-RUNX1 axis in regulating adipocyte differentiation, which warrants further investigation in a separate study. In addition, there was no significant changes observed in the levels of *C/EBPβ* among three different cells.

Together, these data demonstrate that *K43M* precursors derived from SVF had defects in differentiation into white adipocytes, and that deletion of RUNX1 in *K43M* precursors rescued the defects, indicating that RUNX1 mediates the effect of CDK6 in adipocyte differentiation in a cell autonomous manner.

## 4 Discussion

Stem cell-related therapies hold great promise for the treatment of many obesity-related metabolic diseases, however, the mechanisms of regulation of adipocyte stem cells are unclear at present. In this report, we have found that mice carrying a kinase inactive *Cdk6* allele (*K43M*) had a pronounced reduction in the number of adipocyte precursors. In contrast, mice carrying the INK4-insensitive, hyperactive *Cdk6* allele (*R31C*) displayed an increase in the number of adipocyte precursors. Reduced precursor proliferation in the absence of CDK6 activity, which limits precursor expansion, is at least one of the reasons for the decreased adipocyte precursor number in *K43M* mice. Evidence presented in this paper indicate that CDK6 positively regulates adipocyte precursors in part through kinase-mediated suppression of RUNX1, since knockout of *RUNX1* in *K43M* mice rescued most defects in both precursors and differentiated cells.

We found that CDK6 regulates white adipocyte precursor differentiation in a cell-autonomous manner. The loss of function of precursors in *K43M* mice is physiological relevant as we have shown previously that loss of CDK6/kinase activity in mice resulted in weight loss, reduced VAT mass, improved glucose tolerance, and insulin sensitization (Hou et al., 2018). Now we showed that gain of function of precursors in *R31C* mice resulted in weight gain, increased VAT mass (Figure 1). The finding that re-expression of CDK6 in adipocyte precursors reversed the differentiation defect in *K43M* cells or knock-down of CDK6 in 3T3-L1 cells copied the phenotypes of *K43M* in proliferation and differentiation (Figures 4, 5) not only confirmed the specificity of CDK6 in regulating adipocyte differentiation, but also demonstrated that it acts in a cell-autonomous manner.

We also demonstrated that the effects of CDK6 in the regulation of adipocyte precursor is dependent on the activity of RUNX1, one of the downstream effectors of CDK6 (Biggs et al., 2006) known to be the most critical regulator of HSC formation in the major vasculature of the mouse embryo (Nottingham et al., 2007) and an essential regulator for MSC proliferation and myofibroblast differentiation (Kim et al., 2014). In our previous studies, we have demonstrated a molecular interplay between CDK6 and RUNX1 in the regulation of white fat browning (Hou et al., 2018) and in the commitment of precursors to beige cell differentiation in SAT (Kim et al., 2014). This mechanism appears, at least in part, to be due to a CDK6 kinase-mediated suppression of RUNX1 which is required for the initiation of precursor commitment to differentiation of white fat but not for beige cell in SAT (Hou et al., 2018), whereas loss of CDK6 kinase activity results in stabilization and recruitment of RUNX1 to the proximal promoter regions of *Ucp-1* and *Pgc-1α(19) and* subsequently leads to increased level of BAT-specific protein expression (Hou et al., 2018), which is associated with a protection against obesity and metabolic diseases in rodent models and correlated with leanness in human (Cederberg et al., 2001; Seale et al., 2011). Our results suggested that the absence of CDK6 kinase activity restricts adipocyte precursors from executing WAT adipogenic programs in a cell-autonomous manner, and that CDK6 is essential for WAT vs*.* BAT lineage commitment.

In the VAT, loss of CDK6 results in a reduction of WAT mass not through white fat browning (Hou et al., 2018), but partially through inhibiting the production of adipogenic precursors and through suppressing their proliferation and differentiation. It is generally accepted that C/EBPα and PPARγ are considered two primary transcriptional factors involved in the initiation of adipogenesis in mouse pre-adipocytes, since adipogenesis does not occur in either C/EBPα- or PPARγ-deficient MEFs (Barak et al., 1999; Linhart et al., 2001). Both C/EBPα and PPARγ are regulated by C/EBPβ through association with C/EBP regulatory elements within the promoters of the corresponding genes (Wu et al., 1995; Clarke et al., 1997).

CDK6 serves as the principal kinase *in vivo* mediating RUNX1 phosphorylation which increases the transactivation potency of RUNX1 (Zhang et al., 2008). In addition, RUNX1 interacts with several cell lineages C/EBP transcription factor family members to jointly bind and activate transcription of target genes. For instance, RUNX1 functions in concert with lineage-specific transcriptional factor C/EBPα in granulopoiesis (Fujimoto et al., 2007), whereas CDK6 blocks myeloid differentiation by interfering with RUNX1 DNA binding and interaction between RUNX1 and C/EBPα (Fujimoto et al., 2007); RUNX1 interact with C/EBPβ transcription factor to coordinately activate transcription of adipogenic target genes (Ugarte et al., 2012). Indeed, we found that loss of CDK6 protein (Figure 5)/kinase activity (Figure 4; Figure 8) resulted in reduction of mRNA levels of C/EBPα, PPARγ, and PPARα, and ablation of *Runx1* in *K43M* mice increased expression of mRNA levels, accompanied by the rescue of the defect in WAT development in mature adipocytes *in vitro* (Figure 8) and *in vivo* (Hou et al., 2018). Of note, upon deletion of Runx1 in *K43M* cells, the expression of *Fabp4* and *Adiponectin* was dramatically increased over and above that of *WT*, suggesting that either a synergistic effect of CDK6 and RUNX1 or the involvement of other molecules and mechanisms in the CDK6-RUNX1 axis in regulating adipocyte differentiation, which warrants further investigation in a separate study.

However, our understanding of the molecular mechanism for CDK6 regulation in the stem and progenitors derived from adipose tissues is limited at present. Several key questions remain unanswered. (Brooks et al., 2018). What are the molecular mechanisms employed by CDK6 in promoting the production of precursors in addition to proliferation (Bhansali et al., 2017)? What are the genes regulated by CDK6-RUNX1 that play key roles in self-renewal, survival, and proliferation of precursors? Future studies addressing these questions will provide novel insights, which can be translated to therapeutic approaches.

Overall, our findings reveal that CDK6 kinase activity functions as a potent regulator of adipocyte stem cell proliferation and differentiation, providing a new therapeutic target for pharmacological intervention aimed at combatting the imminent obesity epidemic and its related metabolic diseases.

## Data Availability

The original contributions presented in the study are included in the article/Supplementary Material, further inquiries can be directed to the corresponding author.
