# Supplementary material for: CDK6 is essential for mesenchymal stem cell proliferation and adipocyte differentiation
Source: Front Mol Biosci. 2023 Aug 16;10:1146047. doi: 10.3389/fmolb.2023.1146047 (PMC10469316; doi:10.3389/fmolb.2023.1146047)
Supplement: Supplementary file 1 [file DataSheet1.pdf]

Supplementary Figures and Table

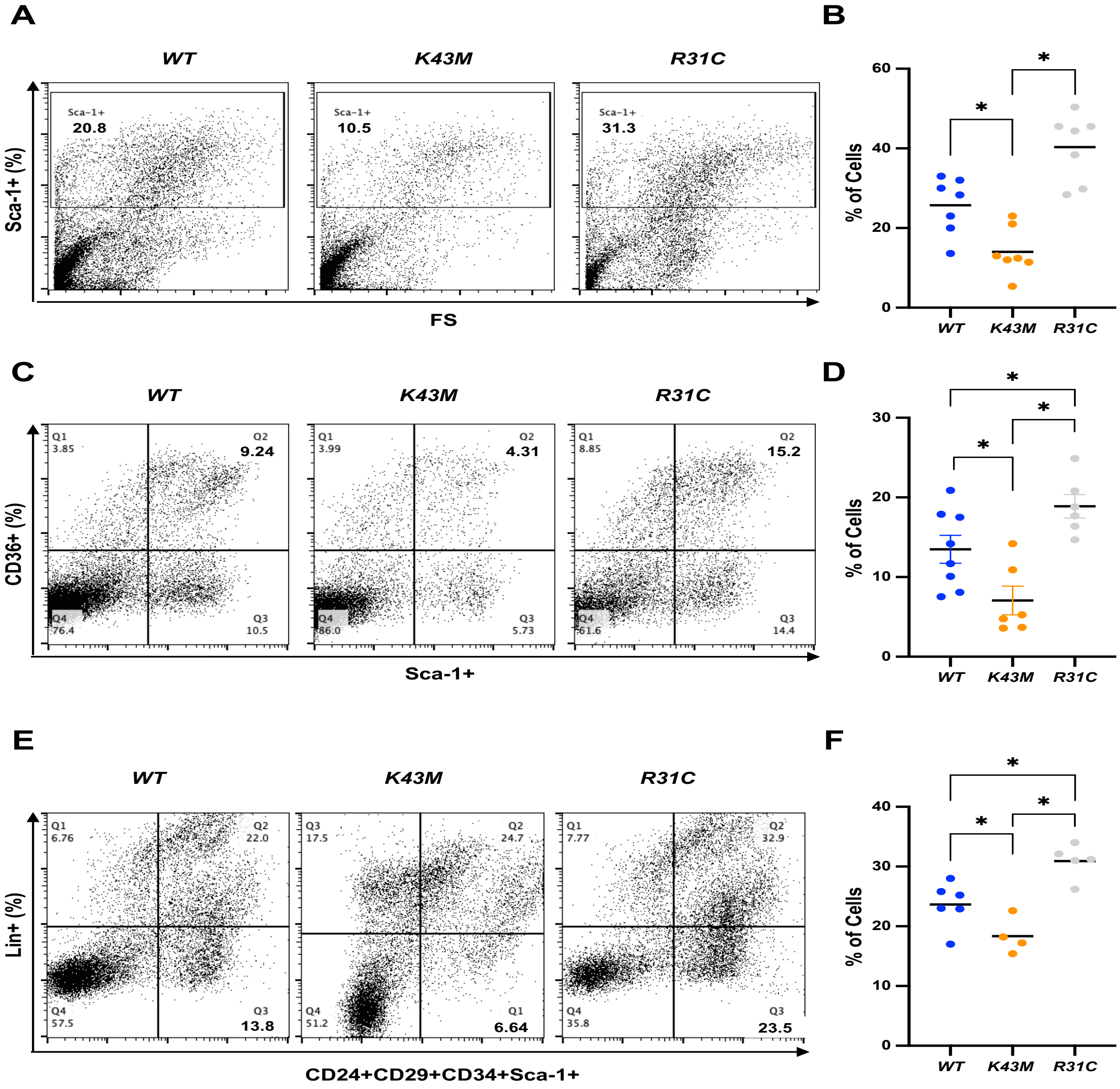

Supplementary Figure 1: *Reduced adipocyte precursors in female K43M mice and increased adipocyte precursors in female R31C mice.* **(A, C, E)** Representative flow cytometric profiles of Sca-1$^+$(**A**), Sca-1$^+$CD36$^+$(**C**), and Lin$^-$Sca-1$^+$CD24$^+$CD29$^+$CD34$^+$ (**E**) cells isolated from eWAT of female *WT*, *K43M, and R31C* mice at 18-20 weeks of age. (**B, D, F**) Histograms summarizing the Sca-1$^+$ (**B**), Sca-1$^+$CD36$^+$(**D**), and Lin$^-$Sca-1$^+$CD24$^+$CD29$^+$CD34$^+$ (**F**) cells in panel **A, C,** and **E**, respectively. For **B, D,** and **F,** data shown are mean ± SE (*n* = 6-10). *p < 0.05, t-test.

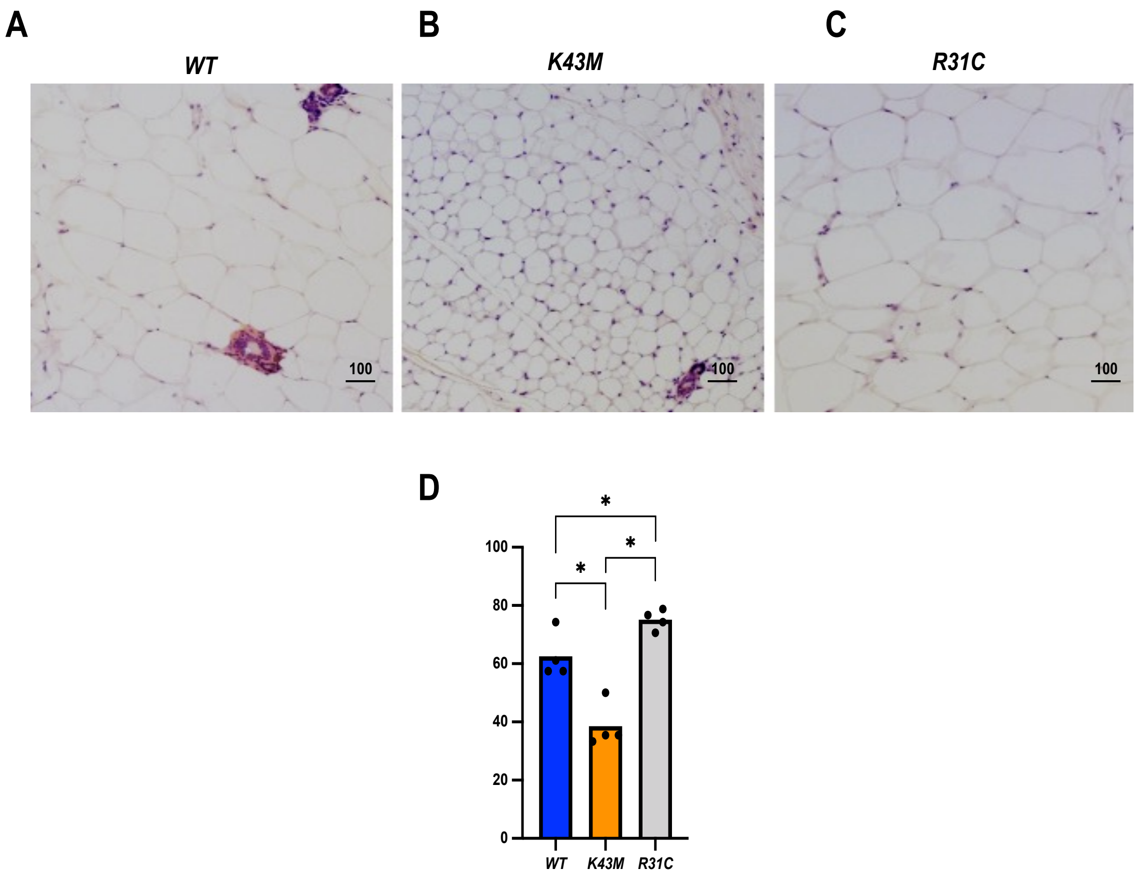

Supplementary Figure 2. Loss of CDK6 kinase activity in mice resulted in reduced the cell size of mature adipocytes and decreased proliferation of precursors. (A-C) Representative light microscopic images of H&E-stained sections of eWAT from male mice indicated (n=4 for each genotype) (scale bars: 100 μm). (**D**) Bar graph summarizing the percentage of double positive cells for DAPI (blue) and BrdU (green) per field under microscopy in Figure 3A-C. Data shown are mean ± SE (n = 4). *$p < 0.05$, t-test.

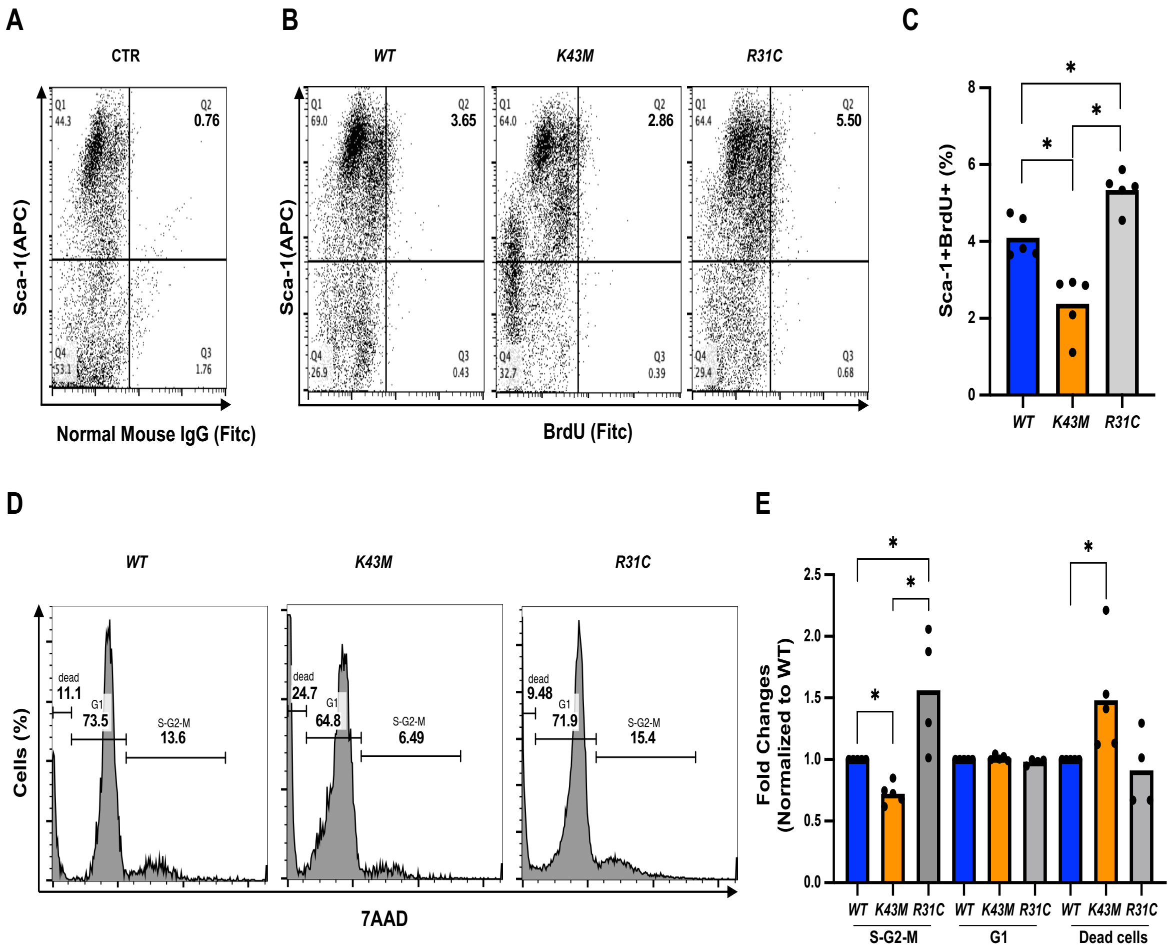

Supplementary Figure 3. ***Role of CDK6 kinase activity in BrdU incorporation and cell cycle profiles.*** (**A-C**) Representative flow cytometric profiles of negative control with SVF cells stained with normal mouse IgG-FITC and Sca-1-APC (**A**) and Sca-1^+BrdU$^+$ cells isolated from eWAT of female *WT*, *K43M, and R31C* mice at 18-20 weeks of age (**B**). (**C**) Histograms summarizing the Sca-1^+BrdU$^+$ cells in panel **B**. (**D**) Representative flow cytometric profile profiles of Sca-1^+7AAD$^+$ cells isolated from eWAT of female *WT*, *K43M, and R31C* mice at 18-20 weeks of age. (**E**) Histograms summarizing the Sca-1^+7AAD$^+$ cells in panel **D**. For C**,** data shown are mean ± SE (n = 5), *$p < 0.05$, t-test. For **E**, data shown are fold change of cells in different cell cycle phases normalized to the relative *WT* controls, which was arbitrarily defined as 1 unit. Data shown are mean ± SE (n = 4-5). *$p < 0.05$, t-test.

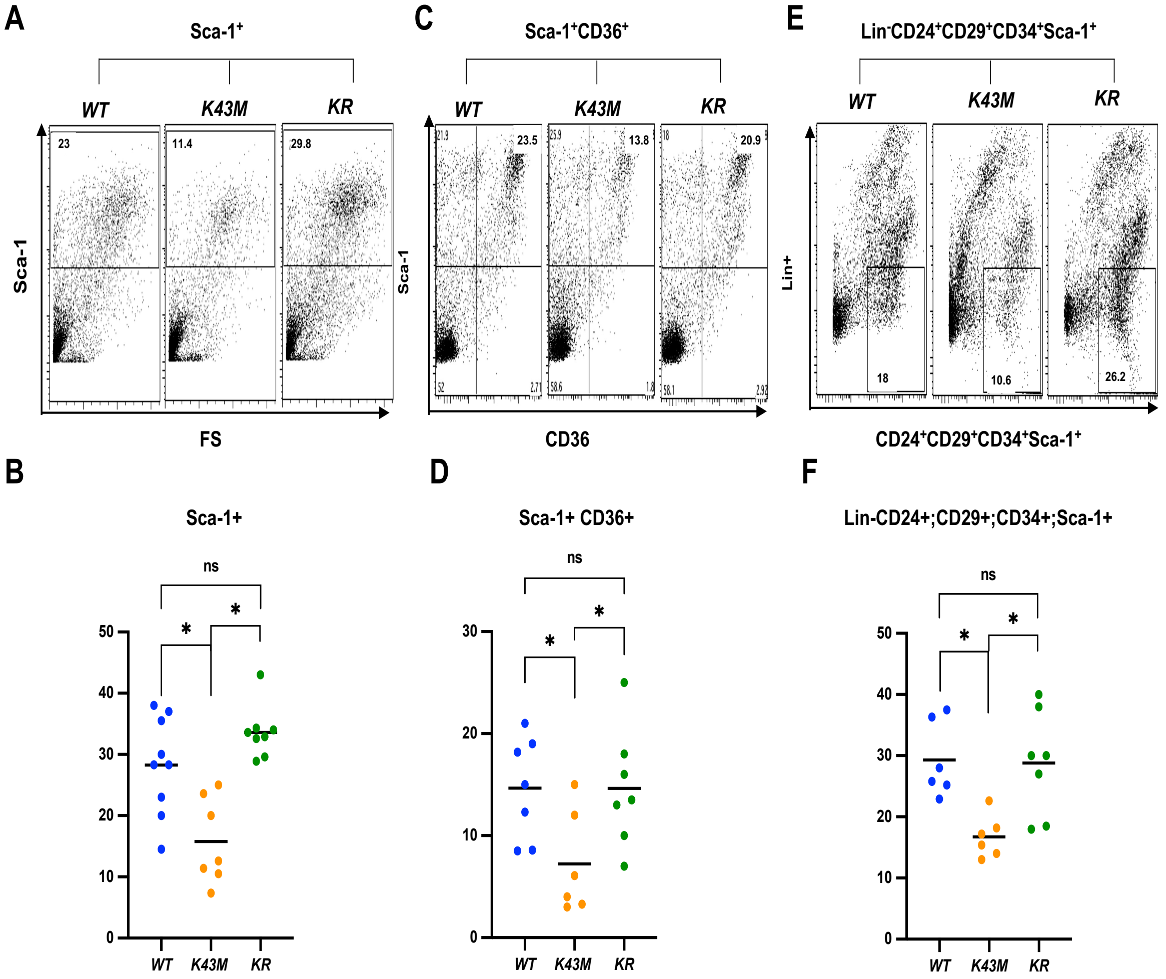

Supplementary Figure 4. ***Ablation of RUNX1 in mature adipocytes rescued the defect of precursor numbers in K43M mice.*** **(A, C, E)** Representative flow cytometric profiles of Sca-1$^+$ (**A**), Sca-1$^+$CD36$^+$ (**C**), and Lin$^-$Sca-1$^+$CD24$^+$CD29$^+$CD34$^+$ (**E**) cells isolated from eWAT of female *WT*, *K43M, and KR* mice at 4-5 months of age. (**B, D, F**) Histograms summarizing Sca-1$^+$ cells in panel **A (B),** Sca-1$^+$CD36$^+$ cells in panel **C** (**D**), and Lin$^-$Sca-1$^+$CD24$^+$CD29$^+$CD34$^+$ cells in panel **E** (**F**). For **B, D,** and **F,** data shown are mean ± SE (n = 6-9). *$p < 0.05$, t-test.

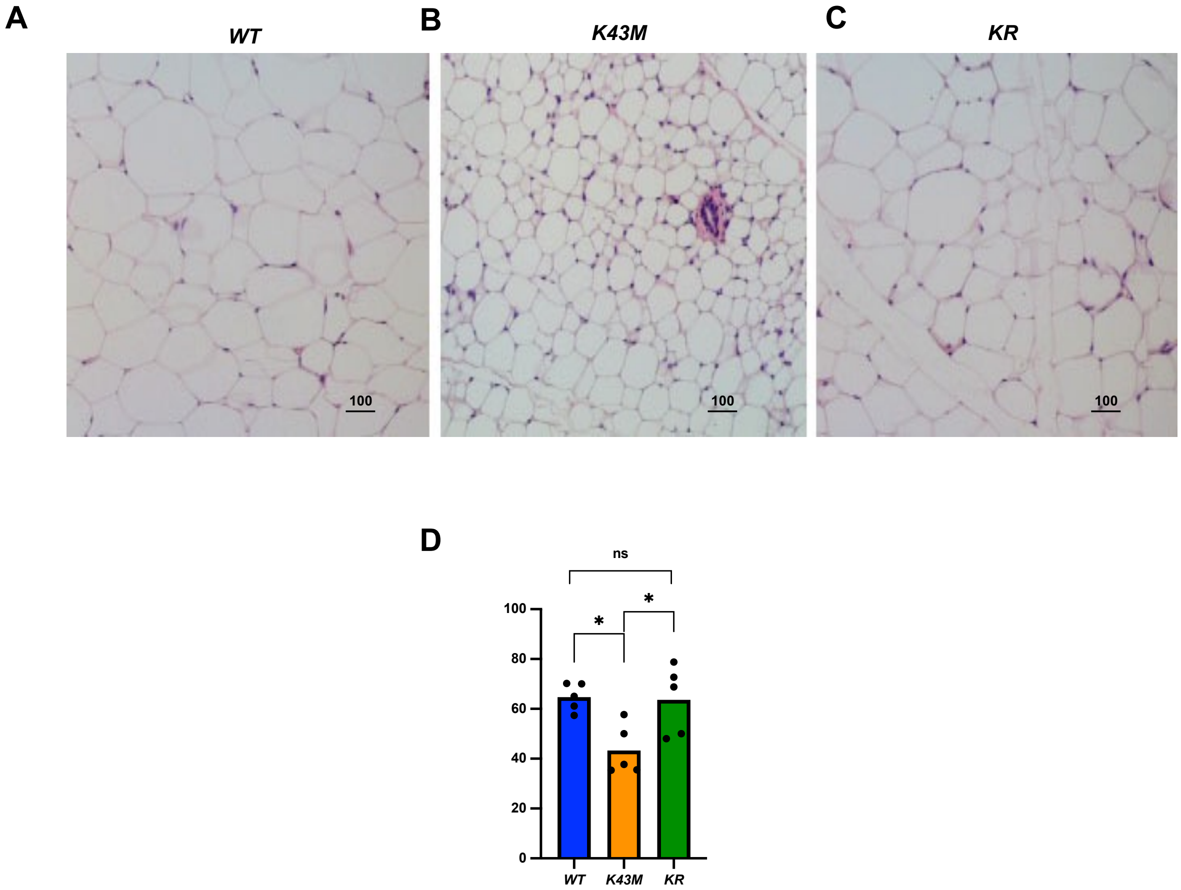

Supplementary Figure 5. ***Ablation of RUNX1 in K43M mature adipocytes reversed the cell size and increased BrdU incorporation.*** **(A-C)** Representative light microscopic images of H&E-stained sections of eWAT from male mice indicated (n=4 for each genotype) (scale bars: 100 μm). (**D**) Bar graph summarizing the percentage of double positive cells for DAPI (blue) and BrdU (green) per field under microscopy in Figure 7 A-C. Data shown are mean ± SE (n = 4). $^{*}p < 0.05$, t-test.

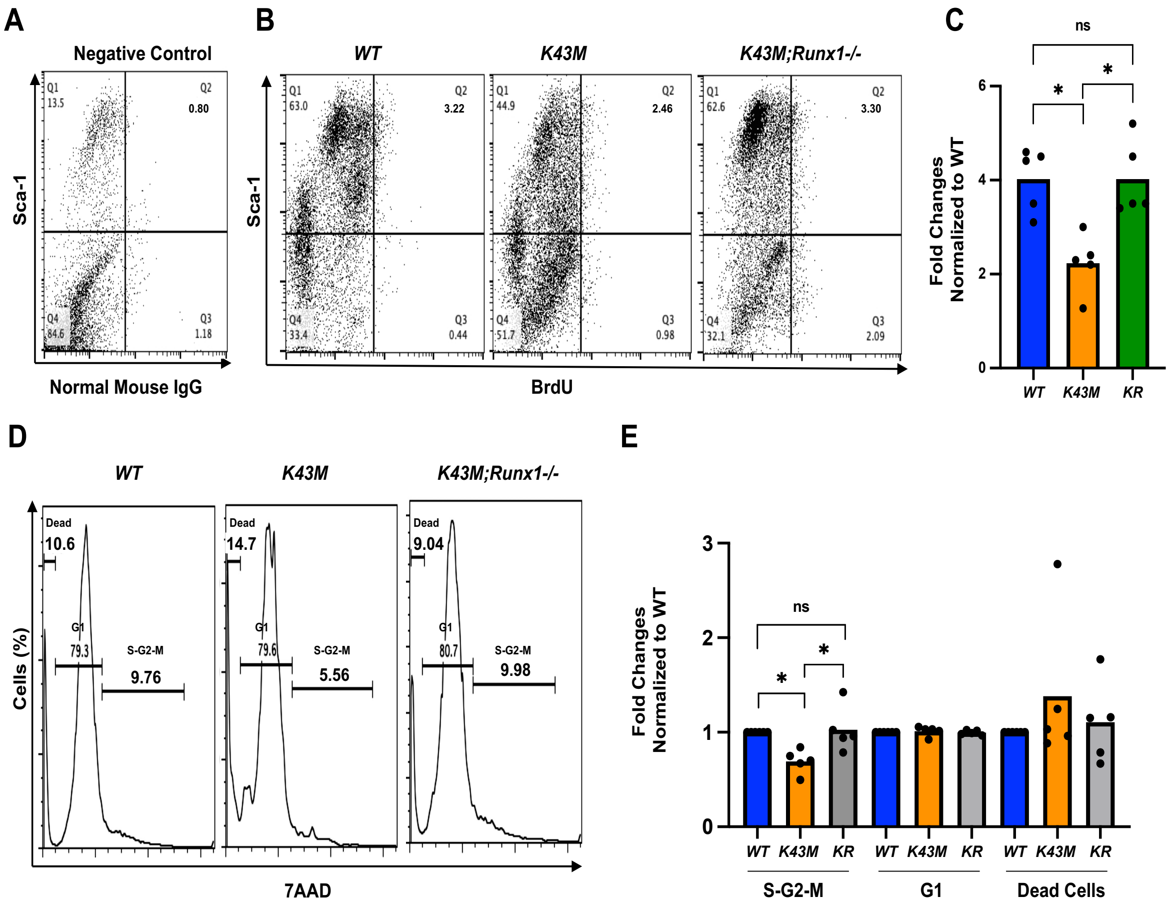

Supplementary Figure 6. ***Ablation of RUNX1 in K43M mature adipocytes increased BrdU incorporation, proliferation, and decreased dead cells in K43M cells.*** (**A-B**) Representative flow cytometric profiles of negative control with SVF cells stained with normal mouse IgG-FITC and Sca-1-APC (**A**) and Sca-1^+BrdU$^+$ cells isolated from eWAT of female *WT*, *K43M, and KR* mice at 4-5 months of age (**B**). (**C**) Histograms summarizing the Sca-1^+BrdU$^+$ cells in panel **B**. (**D**) Representative flow cytometric cell cycle profiles of Sca-1^+7AAD$^+$ cells isolated from eWAT of female *WT*, *K43M, and KR* mice at 4-5 months of age. (**E**) Histograms summarizing the Sca-1^+7AAD$^+$ cells in panel **D**. For **C,** data shown are mean ± SE (n = 5), *$p < 0.05$, t-test. For **E**, data shown are fold change of cells in different cell cycle phases normalized to the relative *WT* controls,

which was arbitrarily defined as 1 unit. Data shown are mean ± SE (*n* = 4-5). *$p < 0.05$, t-test

Table 1, Primers sequences used for quantitative real-time PCR.

Primer names	Sequences (5' to 3')
m-C/EBPα-F	CAAGAACAGCAACGAGTACCG
m-C/EBPα-R	GTCACTGGTCAACTCCAGCAC
m-C/EBPβ-F	CGCCCGCCGCCTTTAGAC
m-C/EBPβ-R	CGCTCGTGCTCGCCAATGG
m-PPARα-F	AGGAAGCCGTTCTGTGACAT
m-PPARα-R	TTGAAGGAGCTTTGGGAAGA
m-PPARγ-F	ATGGGTGAAACTCTGGGAGATTCT
m-PPARγ-R	CTTGGAGCTTCAGGTCATATTTGTA
